# A comprehensive proteomics-based interaction screen that links DYRK1A to RNF169 and to the DNA damage response

**DOI:** 10.1038/s41598-019-42445-x

**Published:** 2019-04-12

**Authors:** Julia Roewenstrunk, Chiara Di Vona, Jie Chen, Eva Borras, Chao Dong, Krisztina Arató, Eduard Sabidó, Michael S. Y. Huen, Susana de la Luna

**Affiliations:** 1 Centre for Genomic Regulation (CRG), The Barcelona Institute of Science and Technology (BIST), 08003 Barcelona, Spain; 2 Centro de Investigación Biomédica en Red en Enfermedades Raras (CIBERER), Barcelona, Spain; 3 School of Biomedical Sciences, LKS Faculty of Medicine, The University of Hong Kong, S.A.R. Hong Kong, China; 4 Universitat Pompeu Fabra (UPF), 08003 Barcelona, Spain; 5 State Key Laboratory of Brain and Cognitive Sciences, The University of Hong Kong, S.A.R. Hong Kong, China; 6 Institució Catalana de Recerca i Estudis Avançats (ICREA), 08010 Barcelona, Spain

## Abstract

Dysregulation of the DYRK1A protein kinase has been associated with human disease. On the one hand, its overexpression in trisomy 21 has been linked to certain pathological traits of Down syndrome, while on the other, inactivating mutations in just one allele are responsible for a distinct yet rare clinical syndrome, DYRK1A haploinsufficiency. Moreover, altered expression of this kinase may also provoke other human pathologies, including cancer and diabetes. Although a few DYRK1A substrates have been described, its upstream regulators and downstream targets are still poorly understood, an information that could shed light on the functions of DYRK1A in the cell. Here, we carried out a proteomic screen using antibody-based affinity purification coupled to mass spectrometry to identify proteins that directly or indirectly bind to endogenous DYRK1A. We show that the use of a cell line not expressing DYRK1A, generated by CRISPR/Cas9 technology, was needed in order to discriminate between true positives and non-specific interactions. Most of the proteins identified in the screen are novel candidate DYRK1A interactors linked to a variety of activities in the cell. The in-depth characterization of DYRK1A’s functional interaction with one of them, the E3 ubiquitin ligase RNF169, revealed a role for this kinase in the DNA damage response. We found that RNF169 is a DYRK1A substrate and we identified several of its phosphorylation sites. In particular, one of these sites appears to modify the ability of RNF169 to displace 53BP1 from sites of DNA damage. Indeed, DYRK1A depletion increases cell sensitivity to ionizing irradiation. Therefore, our unbiased proteomic screen has revealed a novel activity of DYRK1A, expanding the complex role of this kinase in controlling cell homeostasis.

## Introduction

The dual-specificity tyrosine phosphorylation-regulated kinase (DYRK) family of serine/threonine protein kinases belongs to the CMGC group, and it is present in all eukaryotes^1,2^. Based on their phylogenetic relationships, DYRKs are divided into three subfamilies: PRP4s, HIPKs and DYRKs. In turn, the DYRK subfamily is divided in Yak-type kinases, and class I or class II DYRKs. In humans, there are five members of the DYRK subfamily: DYRK1A and DYRK1B from class I; and DYRK2, DYRK3 and DYRK4 from class II. DYRKs are characterized by their unusual mechanism of activation, whereby autophosphorylation of a tyrosine residue in their activation loop during translation renders the kinase capable of phosphorylating serine and threonine residues^3,4^.

Given its links to human disease, DYRK1A is the best-known member of the family. The three copies of its encoding gene in trisomy of chromosome 21 provoke a 1.5-fold overexpression. This excess of DYRK1A has been implicated in several pathological traits of Down syndrome, including the increased risk of childhood leukaemia, skeletal abnormalities, intellectual disability, motor coordination and retinal defects^5–10^. By contrast, inactivating mutations in just one *DYRK1A* allele (gene truncation, small deletions and insertions, or nonsense mutations) are responsible for a rare syndrome known as DYRK1A haploinsufficiency (OMIM: 614104; ORPHA: 464306), characterized by a general developmental delay, microcephaly, seizures and a characteristic facial gestalt^11^. Moreover, deregulation of the *DYRK1A* gene could also be involved in other human pathologies, such as neurodegenerative diseases, diabetes, osteoporosis or cardiac dysfunction^12–15^, and recent evidence points to a role for DYRK1A in the progression of several types of cancer^16–20^. However, the role of DYRK1A as a negative or positive effector of tumor progression could be complex and tumor cell-dependent. Thus, while inhibiting its kinase activity or dampening its expression hinders the progression of glioblastoma, pancreatic, and head and neck cancer cells^16–18^, the opposite is true for acute myeloid leukemia and breast cancer cell lines^19,20^. Thus, it seems that DYRK1A is probably involved in a variety of molecular and cellular pathways, as reflected by the fact that several of its known substrates and interacting proteins have been connected to different cellular processes^2,21^. However, given the range of phenotypic alterations when this kinase is perturbed, the list of DYRK1A targets, regulators and substrates is expected to keep growing.

Previous proteomic screens based on affinity purification (AP) followed by mass spectrometry (MS) identification involved the overexpression of tagged DYRK1A^22,23^. However, considering that exquisite control of DYRK1A gene dosage is required for its non-pathological activity and the fact that overexpression drives its translocation to the nucleus, such approaches could identify artifactual interactions, highlighting the need to search for interactors under more physiological conditions. Therefore, we addressed this issue by using label-free quantitative MS-based proteomics on DYRK1A purified using specific antibodies, capturing proteins recruited directly or indirectly to the endogenous DYRK1A (i.e., interactors). Most of the proteins identified are novel, candidate DYRK1A interactors, enhancing the complexity of the potential biological functions of DYRK1A. In particular, an in-depth characterization of the functional interaction of DYRK1A with the E3 ubiquitin (Ub) ligase RNF169^24–26^ revealed a role for DYRK1A in the DNA damage response.

## Results

### The DYRK1A protein kinase interactome

**Figure 1:**
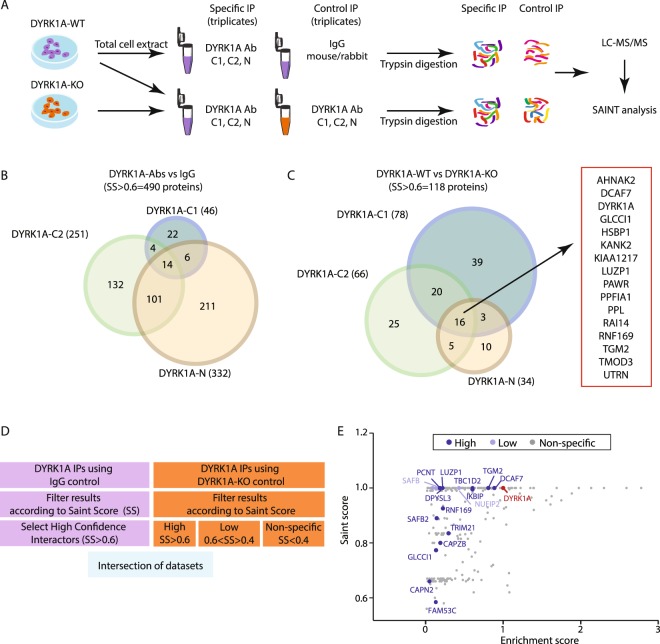
The endogenous DYRK1A interactome in HeLa cells. (**A**) Experimental workflow for the AP-MS experiments. (**B**,**C**) Venn diagrams showing the number and overlap of proteins identified with a SAINT score = 0.6–1 when DYRK1A was immunoprecipitated from total extracts of HeLa cells, with mock immunoprecipitations (**B**) or HeLa-DYRK1A^KO^ extracts (**C**) as the controls. The list of the common interactors among the three antibodies in **C** is included. (**D**) Scheme to filter the non-specific interactions. (**E**) Scatter plot representing the output of filtering the Ab *vs* IgG dataset with the WT *vs* KO dataset. Only proteins found to be “high confidence” interactors in the first set of experiments are represented as dots using their corresponding SAINT score and enrichment score (ES, the mean of the different experiments). The color code indicates the categories from the experiment using HeLa DYRK1A^KO^ cells. The proteins belonging to the “high” or “low” confidence interactor group using DYRK1A^KO^ cells are highlighted in dark and light blue, respectively.

The workflow to identify endogenous direct/indirect interactors of DYRK1A is shown in Fig. 1A and uses, as a source of DYRK1A, soluble cell extracts that mainly reflect the cytosolic pool of DYRK1A (Fig. S1A). DYRK1A purification was attained by using 3 different commercial antibodies (Abs) for DYRK1A: Ab-N, directed against the N-terminus (a rabbit polyclonal antiserum raised against a synthetic peptide corresponding to amino acids [aa] 32–51); and two Abs that recognize epitopes in the C-terminal region of DYRK1A, Ab-C1 (a mouse monoclonal Ab prepared with a glutathione-S-transferase [GST]-DYRK1A fusion peptide of aa 674–763) and Ab-C2 (a rabbit polyclonal antiserum raised against a synthetic peptide corresponding to aa 733–752). All three Abs successfully immunoprecipitated active DYRK1A (Fig. S1B–D). Finally, two different type of controls were used for the discrimination of specific interactions: one set of experiments was performed with IgG control (mouse or rabbit depending on the Ab), and an additional set of experiments was performed with extracts from HeLa cells not expressing DYRK1A (DYRK1A^KO^), generated by targeted deletion of the *DYRK1A* gene using CRISPR-Cas9 technology (Fig. S1E–H). The immunoprecipitations were followed by direct on-bead trypsin digestion and analysis by single liquid chromatography (LC) combined with tandem MS (LC-MS/MS). These experiments were carried out in triplicate and each experiment was repeated at least twice. We used the Significance Analysis of INTeractome (SAINT) algorithm to discriminate between true positives and non-specific interactors^27^. Given the transient nature of some of DYRK1A’s interactions, a SAINT score (SS) above 0.6 was considered to include low affinity binders. In addition, we generated an enrichment score (ES) that normalized the data by adjusting the spectral counts (SpCs) to both the molecular weight of the prey and to the relative DYRK1A enrichment in each experiment (see Methods for a detailed description). This ES allows different experiments to be compared, providing information on the relative abundance of each prey in the DYRK1A complexes, and it serves as an estimate of the stoichiometric relationships between proteins.

SAINT analysis of the experiments using IgG as controls rendered a total of 490 proteins as high confidence candidate interactors (Fig. 1B and Supplementary Dataset 1). However, the experiments using HeLa-DYRK1A^KO^ cells as controls resulted in much fewer proteins with a SS > 0.6 (Fig. 1C and Supplementary Dataset 2). A comparison of the interactors identified with each Ab revealed little overlap among the proteins identified by the three Abs (Fig. 1B,C), which included the known DYRK1A interactor DCAF7^28,29^: DCAF7 showed similar ES scores with any of the antibodies used in the screen with values suggesting a stoichiometric interaction (Fig. S3A). Surprisingly, the comparison of dataset 2 (KO as control) with dataset 1 (IgG as control) revealed that most of the proteins considered to be high confidence interactors using rabbit/mouse IgGs as a control for specificity were actually non-specific interactors when the DYRK1A null mutant extracts were used (Figs 1D,E; see Fig. S2A for some examples), suggesting that the use of IgGs as control in this type of experiments may be a source of false positives, thus leading to erroneous interpretations.

**Figure 2:**
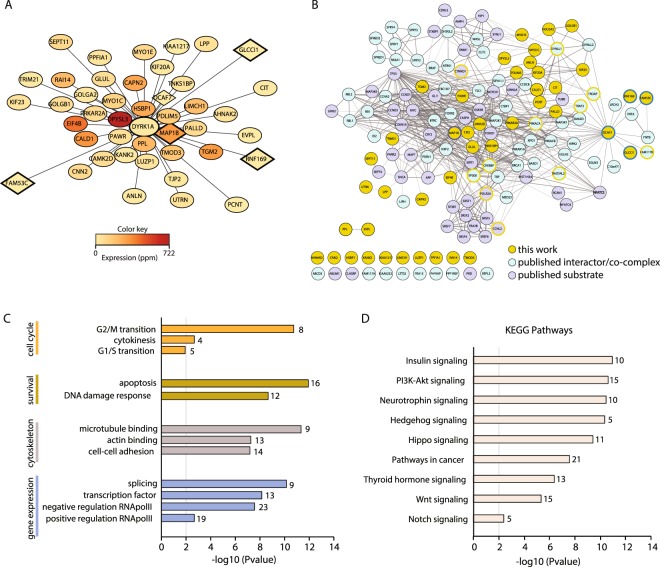
The DYRK1A interactome. (**A**) The high confidence DYRK1A interactors from WT HeLa vs HeLa-DYRK1A^KO^ cells were visualized using Cytoscape: the distance to the bait represents the inverse of the enrichment score (ES, the mean of all AP-MS experiments; the less enriched interactors are located further away from the bait) and the color of the node reflects the abundance of the protein in HeLa cells according to PaxDb (dataset 9606/455). Only proteins identified using at least two antibodies in Fig. 1C are shown. Proteins already known as DYRK1A substrates/interactors are squares. (**B**) Network of all DYRK1A interactors (blue), substrates (violet) and the proteins shown in panel A (orange). The list of DYRK1A interactors and substrates was curated manually (Supplementary Dataset 3). The DCAF7 dataset of interactors was obtained from BioGRID. The network was visualized using Cytoscape and the edge thickness and grey scale indicate the confidence level of the data based on the combined STRING score. Proteins identified in our screen but previously found as interactors or substrate were circled in blue or violet, respectively. Proteins in the network that were found in our screen below the established thresholds are circled in orange. (**C**,**D**) Analysis of the DYRK1A network for Gene Ontology enrichment (**C**) and KEGG signaling pathways (**D**) according to the DAVID software. The graphs show p-values (y-axis) and the number of proteins in each set.

Based on the previous results, we therefore only considered the proteins identified as interactors in this second screen for further analysis. A representation of the proteins complexed with DYRK1A based on both the ES and their relative abundance in HeLa cells shows that there is no direct relationship between the two parameters (Fig. 2A). Thus, it seems that the data are not biased towards abundant proteins, probably reflecting genuine interactions. In addition, most of the interactors identified in the screen are described as cytosolic proteins or found associated to the cytoskeleton according to UniProt annotations (Fig. S2B).

Combining literature mining and information from the BioGRID database, we compiled a list of DYRK1A substrates and/or interactors from both low and high throughput experiments (Supplementary Dataset 3) and we used it for comparison with our MS dataset. Only DCAF7, FAM53C, GLCCI1, PDLIM5 and RNF169 had been identified in previous screens^22,23,30^, while MAP1B had been reported as a DYRK1A substrate^31^ and the CAPN2 paralog calpain 1/CAPN1 has been shown to proteolytically digest DYRK1A^32^. In addition, the DYRK1A substrates p120-catenin/CTNND and cyclin L2/CCNL2^33,34^ were present in the specific DYRK1A immunoprecipitates, although they failed to pass the threshold of specificity established here. Other interactors were only identified in nuclear extracts, such as FAM117B, PRKACA, Arip4/RAD54L2, TRAF3 or TROAP (data not shown) and finally, proteins like CBP/CREBBP or p300/EP300 were identified as low affinity binders in HEK-293T cells alone (data not shown). The limited overlap of our AP-MS data with that already published could reflect the cell-type specificity of the interactions, the experimental approach used to identify the interactions (yeast-two hybrid, pull-down, co-immunoprecipitation/immunoblotting) or the use of exogenously/endogenous expressed proteins. However, the global network derived from the list of DYRK1A interactors has a high degree of interconnectivity (Fig. 2B), highlighting the integration of some of the novel interactors found in our screen with previously identified proteins in the network. Notably, a populated subnetwork was evident around glycogen synthase kinase-3 (GSK3; Fig. S2C) that includes several GSK3 substrates and that concurs with the proposed activity of DYRK1A as a GSK3 priming kinase^2^. Together, the set of DYRK1A substrates and of direct/indirect interactors defines a complex panorama of DYRK1A’s biological roles, with a particular enrichment of activities related to the regulation of cell cycle, gene expression (transcription, splicing) and cytoskeleton-related activities (Fig. 2C). Finally, an analysis of the KEGG pathways showed enrichment of a wide range of signaling pathways (Fig. 2D), which probably contributes to the functional pleiotropism of DYRK1A.

### The E3-ubiquitin ligase RNF169 is a novel interacting partner of DYRK1A

From the list of potential novel DYRK1A interactors, we further explored the functional interaction with the E3 Ub ligase RNF169, because this protein appeared as a high confidence interactor in the DYRK1A screen (Figs 1E and 2A), and because DYRK1A was detected in RNF169 AP-MS experiments^35^. Current evidence situates RNF169 as a key component of the cellular response to DNA double-strand breaks (DSBs)^24–26^, limiting the activity of the p53-binding protein 1 (53BP1)^36^; of note, DNA damage was one of the categories enriched in the DYRK1A interactome (Fig. 2C).

**Figure 3:**
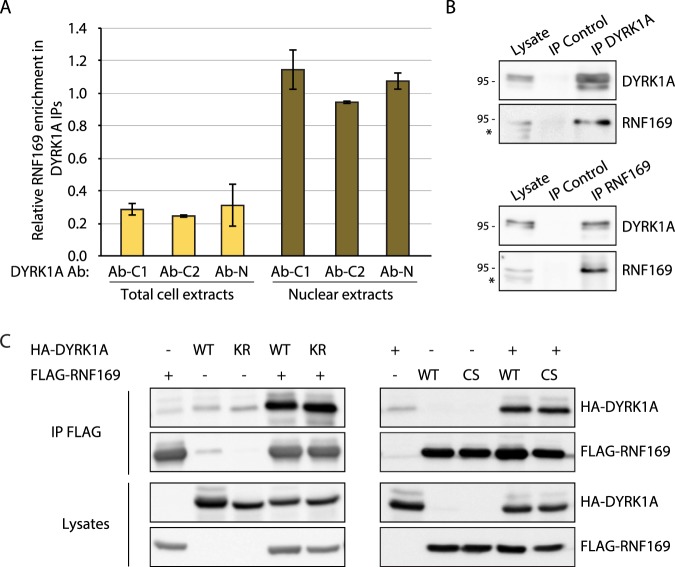
RNF169 mainly interacts with DYRK1A in the nucleus. (**A**) Relative enrichment of RNF169 in total cell extracts (light orange) and nuclear extracts (dark orange) of HeLa cells in the DYRK1A interactome analysis. The graph shows the enrichment score (ES) for each Ab and cellular compartment (mean ± SEM of two independent experiments). (**B**) DYRK1A and RNF169 co-immunoprecipitation experiments using HNEs and Abs against DYRK1A (Ab-C1) or RNF169 (a rabbit IgG was used as a control); *non-specific band. (**C**) HEK-293T soluble extracts transiently expressing the proteins indicated were immunoprecipitated with an anti-FLAG Ab (KR, DYRK1A K188R catalytically inactive mutant; CS, RNF169 C68S inactive mutant). The presence of the proteins was detected in WBs probed with anti-FLAG or anti-HA.

Analysis of the different AP-MS experiments showed that the enrichment of RNF169 is independent of the Abs used to immunoprecipitate DYRK1A, and that RNF169 was much more strongly enriched in HeLa nuclear extracts (HNE) than in the total cell extracts (Fig. 3A), in comparison with the behavior of DCAF7, which appeared similarly enriched in both compartments (Fig. S3A). Given that RNF169 localizes to the nucleus of cells grown in normal conditions^24^, the differential enrichment is probably due to the subcellular distribution of RNF169 rather than any differences in the interaction. The interaction between the two proteins was validated by co-immunoprecipitation experiments in HNE (Fig. 3B), reinforcing that DYRK1A and RNF169 form a complex in the nucleus. The specificity of this interaction was further supported by the failure of DYRK1A to associate with the RNF169 paralog, RNF168 (Fig. S3B).

The direct DYRK1A and RNF169 interaction was confirmed in pull-down experiments with purified proteins (Fig. S3C). In addition, the interaction between DYRK1A and RNF169 appears to be independent of the activity of each individual enzyme, as no differences were observed between the wild-type (WT) proteins and the corresponding catalytically inactive versions in co-immunoprecipitation experiments (Fig. 3C). The inactive version of DYRK1A was obtained by introducing the K188R mutation at the ATP binding site, and an inactive RNF169 mutant was generated by disrupting the RING domain structure through the C68S mutation.

**Figure 4:**
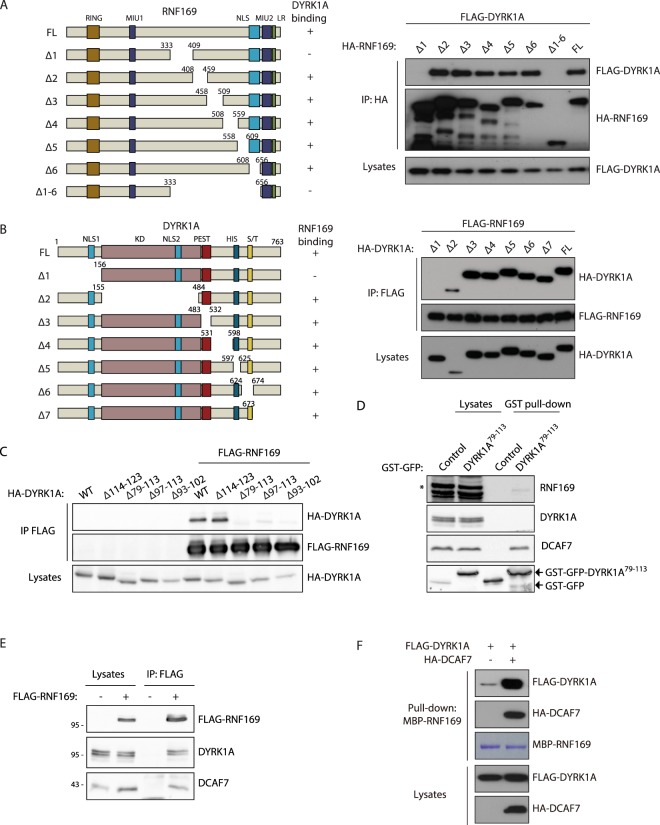
A central region in RNF169 and a conserved region in the N-terminus of DYRK1A are necessary for the DYRK1A-RNF169 interaction. (**A**,**B**) Interaction experiments using HEK-293T cells transiently transfected with the RNF169 and DYRK1A expression constructs indicated, and immunoprecipitated with anti-HA (**A**) or anti-FLAG (**B**) Abs. Schematic illustrations of the RNF169 (**A**) or DYRK1A (**B**) primary structures and the deletion mutants used: HIS, histidine tract; KD, kinase domain; LR, leucine-arginine domain; MIU, motif interacting with ubiquitin; NLS, nuclear localization signal; PEST, PEST region; RING, Really Interesting New Gene domain; S/T, region rich in serine and threonine. (**C**) Soluble extracts from HEK-293T cells transiently expressing the proteins indicated were used in anti-FLAG co-immunoprecipitation experiments (see Fig. S4D for the sequence of the deletions). (**D**) Soluble extracts from HEK-293T cells expressing either aa 79–113 of DYRK1A fused to GST-GFP or GST-GFP alone were subjected to affinity purification using glutathione-Sepharose beads. Both the lysates and the GST-bound fractions were analyzed in WBs probed with Abs to the proteins indicated, and to GFP to detect the fusion proteins: *non-specific band. (**E**) WBs of anti-FLAG immunoprecipitates of HEK-293T soluble extracts expressing FLAG-RNF169. (**F**) Pull-down experiment with MBP-RNF169 expressed in bacteria as bait. HEK-293T extracts expressing FLAG-DYRK1A alone or together with HA-DCAF7 were used as preys, and both the lysates and the bound proteins were analyzed in WBs probed with antibodies to the tags.

The interacting regions of DYRK1A and RNF169 were mapped using a panel of deletion mutants for each of the proteins (Figs 4 and S4). In the case of RNF169, the interacting domain appears to be located in the central region of the protein, mapping to aa 333–409 (Figs 4A and S4A), which does not overlap with any of the known functional domains in the protein. A similar mapping strategy identified the RNF169 binding domain in DYRK1A within the non-catalytic N-terminal domain (Fig. 4B). Refinement of the interaction site with additional DYRK1A mutants framed it between aa 93 and 102, a region necessary and sufficient for this interaction (Fig. 4C,D). Notably, this region coincides with the same minimal binding site for DCAF7^29^ and in fact, this fragment of DYRK1A can recruit endogenous RNF169 and DCAF7 (Fig. 4D). Significantly, DCAF7 was identified in RNF169 immunocomplexes^35^ and validated in co-immunoprecipitation experiments (Fig. 4E), raising the possibility that the three proteins form part of the same protein complex. Indeed, co-expression of DYRK1A and DCAF7 enhanced the interaction between RNF169 and DCAF7 (Fig. 4F), further suggesting that DCAF7 may stabilize the RNF169/DYRK1A complex.

### RNF169 is a DYRK1A substrate

Since RNF169 is an E3 Ub ligase and DYRK1A a kinase^24,26,37^, we wondered whether the physical interaction between the two proteins may result in substrate-dependence. We explored the possibility that DYRK1A was a substrate of RNF169 in two different functional scenarios: (1) catalytic activity, given that non-degradative ubiquitination can regulate protein kinases^38^; and (2) protein stability, considering that DYRK1A ubiquitination affects its half-life^39^. The DYRK1A catalytic activity was not influence by its interaction with RNF169, because DYRK1A N-terminal deletion mutants unable to interact with RNF169 displayed kinase activities that were undistinguishable from the WT protein (Fig. S5A,B). However, we cannot rule out that the interaction of DYRK1A with RNF169 in the correct cell context might still modify its accessibility to substrates and thus, the profile of DYRK1A kinase activity in a specific physiological situation. We also considered whether DYRK1A accumulation might respond to RNF169 activity, but this possibility was ruled out because RNF169 silencing did not affect DYRK1A protein accumulation (Fig. S5C,D). Thus, we conclude that RNF169 is unlikely to be involved in regulating the intrinsic DYRK1A kinase activity or protein levels.

**Figure 5:**
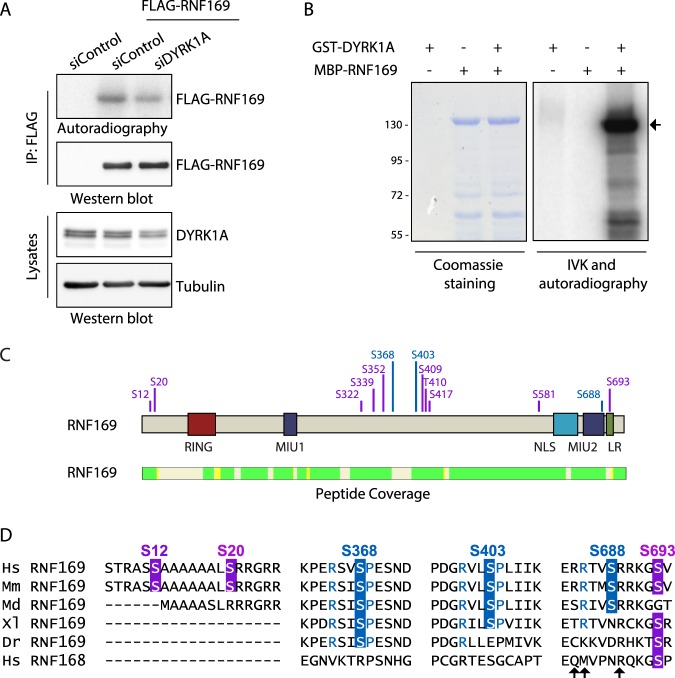
RNF169 is a DYRK1A substrate. (**A**) FLAG-tagged RNF169 expressed in HEK-293T cells depleted of DYRK1A by siRNA transfection was purified using an anti-FLAG Ab and subsequently used in an IVK assay in the presence of radioactive labeled ATP. Proteins were analyzed by autoradiography and in WBs probed with an anti-FLAG Ab to check for equal amounts of RNF169 protein. DYRK1A depletion was assessed in WBs of total lysates from parallel samples. Quantification is shown in Fig. S5E. (**B**) A radioactive IVK assay was performed using bacterially produced MBP-RNF169 in the absence or presence of GST-DYRK1A. The background DYRK1A autophosphorylation was determined by incubation of the protein alone. The Coomassie blue staining demonstrates equal loading and the arrow points to MBP-RNF169. (**C**) MBP-fused RNF169 was used as a substrate in IVK assays with GST-DYRK1A and the phosphorylated peptides were identified by MS analysis. The position of the phosphorylated aa is in violet and the validated residues are in blue. The peptide coverage of RNF169 is also shown (identified peptides in green), rendering a coverage of 87%. See Fig. S6E,F for validation experiments. (**D**) Evolutionary conservation of the DYRK1A-dependent phosphosites in RNF169. Alignment of the RNF169 proteins from different the species and human RFN168: Dr, *Danio rerio*; Hs, *Homo sapiens*; Md, *Monodelphis domestica*; Mm, *Mus musculus*; Xl, *Xenopus laevis*. The phosphosites validated in the IVK assays are shown in blue, with residues at P − 3 and P + 1 in the same color if matching the DYRK1A consensus phosphorylation site. The putative phosphosites assayed in IVK assays but not validated are shown in violet. The arrows indicate residues important for the interaction with the ubiquitinated nucleosome^41^.

We next investigated whether RNF169 is a substrate of DYRK1A by performing radioactive *in vitro* kinase (IVK) assays with FLAG-tagged RNF169 immunoprecipitated from cells in which the activity of DYRK1A was manipulated by means of RNA interference (siRNA transfection). The radioactive signal was above the background in the FLAG-immunocomplexes purified from control cells, suggesting that RNF169 is phosphorylated by co-purifying kinases (Fig. 5A). Notably, the radioactive signal due to the FLAG-purified immunocomplexes obtained from DYRK1A-depleted cells was weaker (Figs 5A and S5E), indicating not only that RNF169 is a phosphoprotein but also that it might be phosphorylated by DYRK1A. The reduction in the radioactive label was even clearer when the experiment was performed in the HeLa-DYRK1A^KO^ cells (Fig. S5F). Indeed, RNF169 was directly phosphorylated by DYRK1A in IVK assays with both DYRK1A and RNF169 purified from bacteria (Fig. 5B).

The identification of the DYRK1A-dependent phosphosites in RNF169 was carried out by MS analysis of MBP-RNF169 phosphorylated by DYRK1A, revealing several putative phosphosites over the entire RNF169 sequence (Fig. 5C). IVK assays using different RNF169 deletion mutants demonstrated that DYRK1A-dependent phosphosites could be found at the N- and C-termini, and in the central region of the protein (Fig. S6A,B), confirming that RNF169 may be phosphorylated by DYRK1A at multiple sites. In addition, we analysed mutants of RNF169 in which specific residues were non-phosphorylatable due to the mutation of serine or threonine to alanine. The sites identified with higher confidence in the MS analyses and/or that adhered to the consensus phosphorylation site described for DYRK1A were chosen (RPXS/TP where X is any aa^40^): S12, S339, S368, S403, S688 and S693. The IVK analysis of the different mutants (Fig. S6C–F) indicated that DYRK1A phosphorylates S368, S403 and S688 in RNF169, at least *in vitro*, while not clear outcome was obtained for S12, S339 and S693 (Fig. 5C). A mutant RNF169 in which the three serine residues were mutated to alanine incorporated less radioactive ATP in IVK assays from cells (Fig. S5F), further supporting the possibility of these sites being target of DYRK1A. The validated phosphosites are evolutionarily conserved or they have undergone conservative substitutions (e.g., replacing the serine residue with aspartic/glutamic acid: Fig. 5D), supporting a functional relevance for these phosphorylation events. The first two phosphosites are located within RNF169 domains of no known function, while S688 is located close to or within the LR-motif^25^ and surrounding residues have been shown to contact ubiquitinated nucleosomes^41,42^.

### DYRK1A is recruited to DNA damage sites by RNF169

**Figure 6:**
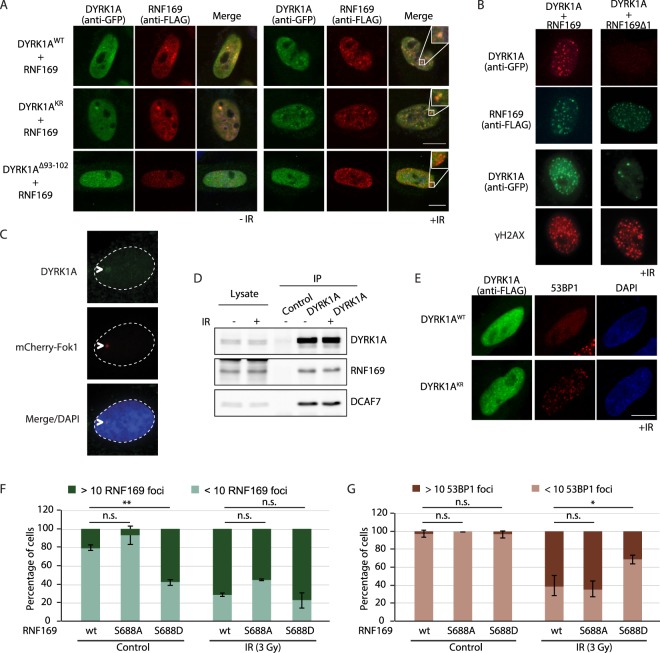
DYRK1A co-localizes with RNF169 at DSBs. (**A**) HeLa cells were transiently transfected with plasmids for the indicated proteins (GFP-DYRK1A and FLAG-RNF169 versions) and 36 h later, they were subjected to IR (3 Gy; +IR) or left untreated (-IR). Cells were fixed after a 1 h recovery and immunostained with the Abs indicated, counter-staining the nuclei with DAPI (blue). Scale bar, 10 µm. Some of the images include an inset to show the overlap in staining. (**B**) HeLa cells expressing FLAG-RNF169 or FLAG-RNF169∆1 (see Fig. 4A) were transfected with a plasmid encoding GFP-DYRK1A. Following 10 Gy irradiation, the cells were pre-extracted using 0.5% Triton X-100 prior to PFA fixation. The cells were subsequently immunolabeled for RNF169 (anti-FLAG) or γH2AX 1 h after recovery, and those cells expressing DYRK1A were detected by direct GFP fluorescence. Only irradiated cells are shown (+IR). (**C**) The U2-OS mCherry-Fok1 reporter cell line was used to detect recruitment of endogenous DYRK1A to DSBs. The DSB site was detected by direct mCherry fluorescence, while the DYRK1A Ab-C1 was used to detect endogenous DYRK1A. Nuclei were counterstained with DAPI. (**D**) DYRK1A was allowed to interact with RNF169 and DCAF7 from untreated or irradiated (3 Gy) HeLa cells and collected in RIPA-buffer 1 h after recovery. (**E**) HeLa cells ectopically expressing FLAG-DYRK1A (WT) or a kinase-mutant version (KR) were irradiated (3 Gy), and subjected to immunofluorescence staining 1 h later with Abs to FLAG (green) and 53BP1 (red). The nucleus of the cells was counterstained with DAPI (blue). Scale bar, 10 µm. (**F**,**G**) HeLa cells expressing the different FLAG-tagged RNF169 proteins were irradiated (3 Gy) and processed for immunofluorescence analysis with anti-FLAG and 53BP1 Abs 1 h after recovery. Cells with more than 10 foci were quantified as positive. The graph shows the percentage of cells positive for RNF169 (**F**) or 53BP1 (**G**) in control cells and after IR (mean ± SEM of two independent experiments). See Fig. S7B for the analysis of the interaction between DYRK1A and the RNF169 proteins.

RNF169 is recruited to DNA damage induced foci upon ionizing irradiation (IR)^24–26^. Therefore, we wondered whether its functional interaction with DYRK1A had an impact on the subcellular localization of the two proteins in response to DNA damage. As previously described for DYRK1A^43^, both DYRK1A WT and a kinase inactive mutant version were localized in the cell nucleus when overexpressed, yet no changes in the subcellular distribution of DYRK1A were observed following irradiation (Fig. S7A). By contrast, DYRK1A adopted a clear punctuate distribution after IR of cells in which it was co-expressed with FLAG-RNF169, and the GFP-DYRK1A foci extensively overlapped those of RNF169 (Fig. 6A). Moreover, the IR-induced foci (IRIF) were also positive for the DNA damage marker γH2AX (Fig. 6B), indicating that DYRK1A and RNF169 co-localize at sites of DNA damage. The recruitment to the DNA damage sites is not an artifact induced by the overexpression of the two proteins, because we have been able to detect endogenous DYRK1A at damage sites using the mCherry-Fok1 cell line, in which DSB is induced at a defined genetic locus (Fig. 6C). Interestingly, the catalytically inactive DYRK1A could also be found at IRIF and it co-localized with RNF169 (Fig. 6A), indicating that the RNF169-mediated recruitment of DYRK1A to IRIF is independent of its kinase activity. Notably, the DYRK1A∆93-102 mutant, which could not interact with RNF169, failed to localize to IRIF after irradiation when co-expressed with RNF169 (Fig. 6A). Likewise, overexpression of the non-interacting RNF169∆1 mutant did not trigger DYRK1A recruitment to IRIF (Fig. 6B). In addition, the interaction between DYRK1A and RNF169 was not affected by IR (Fig. 6D), suggesting that the DYRK1A-RNF169 complex is stable during DSB cellular responses under the conditions used. Together, these results suggest that DYRK1A binding to RNF169 is necessary to recruit DYRK1A to the damaged chromatin.

Given the known role of RNF169 in competing out the binding of 53BP1 to ubiquitinated nucleosomes *in vitro*^41,42^ and to DSB-flanking chromatin *in vivo*^24,25,36^, we tested whether DYRK1A had any impact on this phenotype. Overexpression of DYRK1A, but not that of the kinase dead mutant, suppressed the accumulation of 53BP1 at IR-induced DSBs (Fig. 6E). In view of these results, we focused on the S688 phosphosite in RNF169, located close to the region thought to be important for RNF169 recruitment to IRIF^25^ and for its interaction with ubiquitinated nucleosomes^41,42^. Overexpression of a phosphomimetic RNF169 mutant that affects the S688 site (S688D) resulted in fewer cells with 53BP1 foci when compared to the WT RNF169 (Fig. 6F,G). By contrast, no differences were detected with the mutant that could not be phosphorylated at this residue (S688A: Fig. 6G). These results suggest that phosphorylation of RNF169 at S688 by DYRK1A might modulate RNF169 activity during the DNA damage response.

### Depletion of DYRK1A increases the cell’s sensitivity to DNA damage

**Figure 7:**
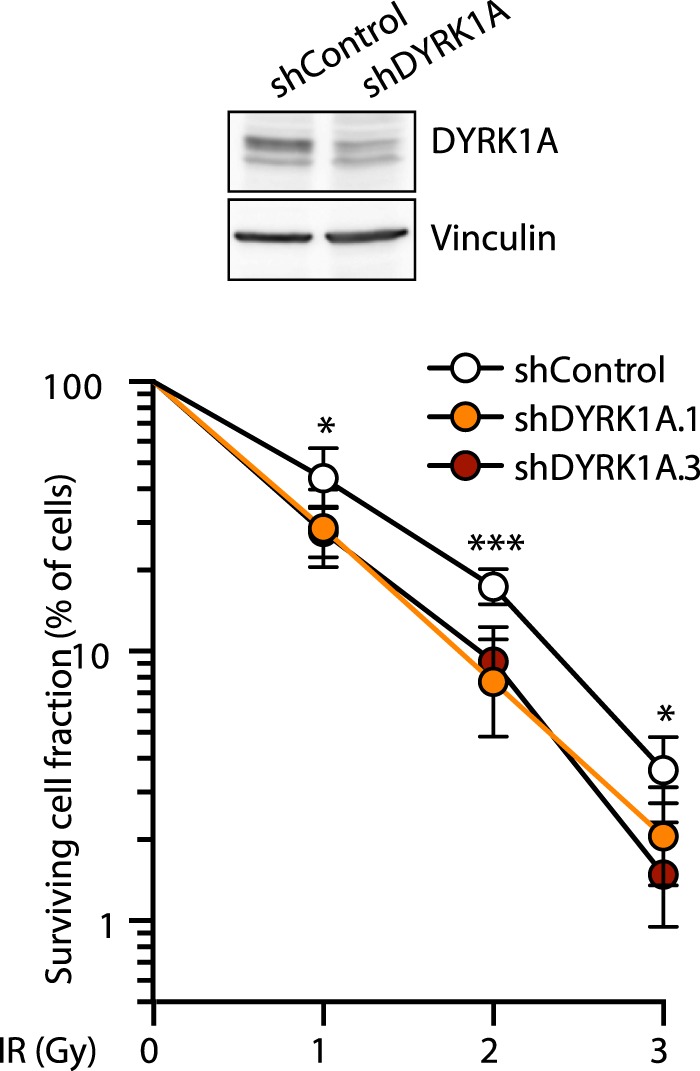
DYRK1A depletion sensitizes cells to DNA damage. Clonogenic survival assay of DYRK1A-depleted cells in response to IR. U2OS cells infected with lentiviruses expressing either non-target shRNA (shControl) or two different shRNAs against DYRK1A were exposed to different IR doses, and the cell’s ability to form colonies was measured after 2 weeks of growth. DYRK1A depletion was assessed in WBs of total cell extracts from parallel samples (a representative experiment is shown). The data correspond to the mean ± SEM (shControl n = 7, shDYRK1A.1 n = 6, shDYRK1A.3 n = 3): *p < 0.05; ***p < 0.001 two-tailed Student’s *t*-test in pairwise comparisons. The Y-axis is represented on a logarithmic scale.

The data presented above led us to ask whether DYRK1A participates in the DNA damage response. To investigate this possibility, we assessed the survival efficiency of irradiated cells following DYRK1A depletion. Results from clonogenic survival assays showed that the proportion of cells that survive DSB induction decreased upon stable knockdown of DYRK1A by lentiviral delivery of shRNAs at all doses tested (Fig. 7). The reduction in long-term survival was also observed in conditions of transient DYRK1A depletion during the IR treatment and recovery (Fig. S8). Hence, cells lacking DYRK1A less effectively recovered from DNA damage when tested in long-term survival assays. Given that the impact on viability, though statistically significant, is small in magnitude, the results could be suggesting that DYRK1A is not acting as a general factor during the DNA damage response, but promoting repair in a subset of IR-induced DSBs that have limited impact on overall cell survival.

## Discussion

In this study, we have used an unbiased AP-MS approach to identify proteins that directly or indirectly associate with the protein kinase DYRK1A under physiological conditions. A significant number of the high confidence DYRK1A interactors found in an initial screen (Ab *vs* IgG control) were found to be non-specific interactors in a second screen on a more representative background (WT cells *vs* KO cells). However, these non-specific interactors are not among the “classic” contaminants commonly observed in AP-MS experiments, since most of them were only weakly or not represented in the CRAPome repository^44^. The results thus highlight that extreme caution needs to be exerted when using mock immunoprecipitations with control IgGs to discriminate between false and true interactors.

Our filtered dataset mostly includes proteins not previously associated to DYRK1A, which at present, cannot be assigned as direct interactors or proteins present in macromolecular complexes with the kinase. Some of them are phosphorylated at serine/threonine residues in the Phosphosite database and with a proline in the +1 position, raising the possibility that they are DYRK1A substrates. The bulk of the DYRK1A interactors found in our screen are mainly cytoplasmic proteins, unlike those obtained in previous AP-MS screens in which the interacting proteins identified were mostly nuclear^22,23^. This difference could reflect a distinct subcellular localization of DYRK1A in the different scenarios, mostly cytosolic under physiological conditions (as seen by cellular fractionation), but accumulating in the nucleus when expressed ectopically, as was the case in the previous screens. Additionally, the list of interactors partially overlap with those identified in a recent screen performed in T98G^30^, suggesting that cell-type specificity might also govern the DYRK1A interactome.

Among the different novel partners, we have functionally characterized the interaction with the E3 ubiquitin ligase RNF169. The domain that interacts with RNF169 was located in the N-terminal non-catalytic region of DYRK1A. The region is evolutionarily conserved, being present not only in DYRK1A orthologs such as *D. melanogaster* minibrain and *C. elegans* MBK-1 but also, in the close paralog DYRK1B (Fig. S4C). Indeed, DYRK1B also appears to associate with RNF169 when they are co-expressed in cells (Fig. S4F), and in AP-MS RNF169 experiments^35^, suggesting that these DYRK family members share functional activities on RNF169. However, the RNF169 interacting region in DYRK1A is not conserved in class II DYRKs, DYRK2, DYRK3 and DYRK4, and accordingly, RNF169 does not appear to interact with these class II DYRKs (Fig. S4E,F). Moreover, this region overlaps with that described to interact with DCAF7^29^, which might indicate that it acts as a docking site for DYRK1A interactors. Although we cannot completely rule out that DYRK1A, RNF169 and DCAF7 interact in pairs, our results are compatible with these three proteins being part of the same complex, which is feasible since the region is predicted to be an amphipathic helix (Fig. S4B). Such a DCAF7-dependent trimeric complex has been already proposed for the interaction of DYRK1A with the adenovirus protein E1A^29^ or with the RNA polymerase II^45^. Notably, DCAF7 participates in E3-Ub ligase complexes^46,47^ and hence, it might facilitate the recruitment of substrates to the DYRK1A-RNF169 complex. On the RNF169 side, the interacting domain has been narrowed to the central part of the protein, which has no associated functions and no homology to its paralog RNF168. This central part of the protein might act as a hinge to separate the RING finger in the N-terminal region from the chromatin-recruitment domains in the C-terminal region, so the binding of DYRK1A should not compete with other RNF169-interacting proteins that bind through these domains. Two of the putative DYRK1A phosphosites in RNF169, S368 and S403, lie within the interacting region. It has been recently reported that a RNF169 protein with these residues mutated to alanine showed reduced capability of interacting with DYRK1A^30^, suggesting that the phosphorylation might work as a regulatory loop by altering the interacting domain.

Our results demonstrate that RNF169 is a DYRK1A substrate, and we have identified three phosphorylation sites in the protein that were all identified previously in high-throughput screens according to Phosphosite; in addition, S368 and S403 have been also identified in a recent publication^30^. The S403 residue was included in the P100 panel to analyze and create phosphoproteomic signatures^48,49^, and it is part of a proteomic signature of drug toxicity in endothelial cells^50^. Notably, phosphorylation of this site is reduced upon cisplatin treatment or IR^51,52^. The C-terminal S688 site is close to the region thought to be important for RNF169 recruitment to the H2A-K13K15ub nucleosomes^41,42^. Notably, recruitment of the RNF169-R689A mutant to DNA damage sites is impaired^25,41^, and residues R684 and R685 are believed to contact DNA in binding assays with nucleosomes containing ubiquitinated H2A^41^. We show that the foci formation capabilities of the phosphomimetic mutant S688D were enhanced in the absence of DNA damage, as if the phosphorylation at this site might influence the recruitment of RNF169 to chromatin. This phenomenon would also explain why the mutant protein behaves more efficiently than WT RNF169 in displacing 53BP1 upon DSB induction.

We also provide clear evidence that DYRK1A binding to RNF169 is necessary for the recruitment of DYRK1A to DSB-induced foci. However, whether phosphorylation of RNF169 occurs at DNA damage sites, and if it is fully dependent on the interaction with DYRK1A are issues that have yet to be determined. Likewise, whether such phosphorylation events only occur in response to DNA damage is another unresolved question, although no changes in DYRK1A kinase activity in response to IR were detected (Fig. S7C,D). Since the DYRK1A/RNF169 complex exists prior to DNA damage, we envisage two possibilities: in one scenario, RNF169 is constitutively phosphorylated and DNA damage triggers its recruitment to such sites; alternatively, RNF169 is not phosphorylated in resting conditions and the DNA damage response is that which creates an appropriate environment at the DSBs for RNF169 phosphorylation. Future experiments will help to determine which of these two possibilities is more likely.

Since DYRK1A has been linked to the DNA damage response through its impact on p53 via phosphorylation of the p53 deacetylase SIRT1^53^, RNF169 could represent another DYRK1A target in this cellular process. From a physiological perspective, the possibility of DYRK1A exerting a role on the cellular response to DNA damage can be contemplated in different scenarios. Deficient responses to DNA damage have been associated to neurodevelopmental defects like microcephaly and neurodegeneration^54^. Significantly, haploinsufficiency of DYRK1A in humans and mice provokes microcephaly^11,55^, whereas DYRK1A overexpression has been associated to neurodegeneration in the adult^12^. It is therefore possible that the inability of brain cells to efficiently cope with DNA damage when DYRK1A expression is dysregulated could contribute to these phenotypes. In a completely different context, the increased sensitivity of cells to DNA damage when DYRK1A is depleted could be therapeutically exploited for cancer treatment by combining DYRK1A inhibitors and genotoxic drugs in cancer types in which DYRK1A inhibitors have an impact on the tumoral cell^16^.

Finally, it is also possible that the functional relationship between DYRK1A and RNF169 extend beyond the DNA damage response. Both proteins are associated with chromatin in the absence of DNA damage^26,56^ and they have been identified in a proteomic screen for interactors of H2B-K120Ub^57^, a chromatin-associated Ub mark mostly related with transcriptional regulation. Hence, there might be some cross-talk between these two proteins at the promoters regulated by DYRK1A, an interesting prospect that we would like to explore in the future.

In summary, we have identified novel DYRK1A partners that widen the potential biological roles of this protein and that will help better understand the molecular pathology of DYRK1A dysregulation. As proof of this possibility, we characterized the functional interaction between DYRK1A and one of its novel targets, placing DYRK1A as an active element in the cellular responses to DNA damage.

## Methods

### Cell culture and cell-based procedures

HeLa, U2OS and HEK-293T cell lines were purchased from the America Type Culture Collection (www.atcc.org). Cells were cultured at 37 °C in Dulbecco’s Modified Eagle’s Medium (Invitrogen) supplemented with 10% fetal bovine serum (FBS, Invitrogen), 100 U/ml penicillin and 100 µg/ml streptomycin (Invitrogen). The cells were transfected using the calcium phosphate precipitation method (HEK-293T) or Lipofectamine 3000 (HeLa: Thermo Fisher Scientific) and they were processed 24–48 h after transfection. The plasmids used for transient transfections are described in the Supplementary Methods. The Lipofectamine^®^ 3000 transfection kit was used to deliver siRNA into the cells, following the manufacturer’s instructions. The siRNAs used were purchased from GE Healthcare Dharmacon: siControl (D-001810-10), siDYRK1A (L-004805-00), siRNF169 (L-032290-00). For lentiviral-dependent transduction of short hairpin (sh)RNAs, pLKO.1-puro-derived vectors were obtained from the Sigma Mission collection: shRNA control, non-targeting vector (SHC001); shRNAs to *DYRK1A*: sh1-TRCN0000022999, sh2-TRCN0000199464, sh3-TRCN0000010613; shRNAs to *RNF169*: TRCN0000265616. Details for the generation of a HeLa cell line with *DYRK1A* genetically inactivated by a CRISPR/Cas9 approach are provided in the Supplementary Methods.

For clonogenic assays, cells transduced with the lentivirus expressing the control shRNA or shDYRK1A were subjected to IR, or they were left untreated before plating at low density. In some experiments, DYRK1A knockdown was obtained by transfection with siRNAs (5′-CGGUCGCUGACUACUUGAAdTdT-3′) twice at 24-h intervals using Oligofectamine (Invitrogen). Cells were allowed to recover for 24 h and, and were either subjected to IR treatment or left untreated before plating at low density. After 7–14 days, the cells were stained with 0.2% methylene blue (Sigma) in 50% methanol and the colonies in triplicate plates were counted. The number of colonies derived from IR-treated cells was normalized to those of untreated cells.

### Immunoprecipitation assays

For immunoprecipitation, soluble cell extracts or HNE (CIL Biotech) were incubated with Magnetic protein G or protein A beads (Invitrogen) bound to specific Abs in binding buffer (50 mM Hepes [pH 7.4], 150 mM NaCl, 2 mM EDTA, 1% Nonidet P-40 [NP-40], a protease inhibitor cocktail [cOmplete Mini, Roche Diagnostic] and phosphatase inhibitors [2 mM Na_3_VO_4_, 30 mM Na_4_P_2_O_7_, 25 mM NaF]). The Abs used were: DYRK1A Ab-C1 (Abnova H00001859-M01); DYRK1A Ab-C2 (Abcam ab69811); DYRK1A Ab-N (Sigma D1694); FLAG (Sigma, F1804); HA (BioLegend, 901501); RNF169 (Bethyl Laboratories, A304-097A); or control immunoglobulin G (rabbit IgGs, Cell Signaling, #2729S; mouse IgGs, Santa Cruz, #sc-2025). The beads were then washed three times with binding buffer, and once with the same buffer without NP-40. The samples were analyzed in Western blots (WBs) or used for IVK assays. Details on the preparation of the cell extracts and WB analysis are provided in the Supplementary Methods.

### *In vitro* kinase assays

For the IVK assays, immunocomplexes or GST-fusion proteins expressed in bacteria (see Supplementary Methods) were incubated for 20 min at 30 °C, in the presence or absence of GST-DYRK1A, in 20 µl of phosphorylation buffer (25 mM Hepes [pH 7.4], 5 mM MgCl_2_, 5 mM MnCl_2_, 0.5 mM DTT) that contained 50 µM ATP and 2.5 µCi [γ-^32^P]-ATP (3,000 Ci/mmol, Amersham Biosciences). The incorporation of ^32^P was determined by SDS-PAGE and exposing the dried gel to film.

DYRK1A kinase activity was assessed using the DYRKtide peptide as the substrate^40^. Briefly, DYRK1A-immunocomplexes were incubated for 20 min at 30 °C in 20 µl of phosphorylation buffer including 200 µM DYRKtide and 2.5 µCi [γ-^32^P]-ATP (3,000 Ci/mmol). Aliquots of the reaction were dotted onto P81 Whatman phosphocellulose paper in triplicate, washed extensively with 5% orthophosphoric acid and counted in a liquid scintillation counter (Beckman Coulter).

### Immunofluorescence

Cells seeded on coverslips were fixed with 4% paraformaldehyde (PFA) for 15 min at room temperature and permeabilized with 0.1% Triton X-100 in phosphate-buffered saline (PBS) for 10 min. When indicated, the cells were extracted with 0.5% Triton X-100 prior to PFA fixation to highlight foci formation. The cells were then blocked for 1 h at room temperature with 10% FBS in PBS, followed by incubation with the primary Abs for 12–16 h at 4 °C (Table S4). After several washes with PBS, the samples were incubated for 1–2 h at room temperature with secondary fluorophore-conjugated Abs (Table S4). The primary and secondary Abs were diluted in PBS containing 1% FBS. Before mounting with Mowiol® 4–88 (Sigma), the cells were stained with 4′,6-diamidino-2-phenylindole (0.2 µg/ml, DAPI) and washed several times with PBS. For the co-localization analysis, 90% glycerol in PBS was used the mounting medium. For the detection of DYRK1A at DSBs, U2OS mCherry-FokI cells^58^ were treated with SHIELD-1 and 4-hydroxytamoxifen for 4 h before processing. A Leica TCS SP5 CFS confocal microscope or a Leica DMI 600B microscope was used to obtain pictures with the Leica Application Suite software. The pictures were analysed with the Fiji image software^59^.

### Mass spectrometry analysis

To identify the proteins in the DYRK1A immunocomplexes, endogenous DYRK1A was immunoprecipitated from 1 mg of total cell lysates (see Supplementary Methods for details on extract preparation) using 10 µg of the specific Ab or mouse/rabbit IgGs as controls. All experiments were performed in triplicate and at least two independent experiments for each Ab were analysed. Following immunoprecipitation, the beads were washed three times with 500 µl of 200 mM NH_4_HCO_3_ and then, 60 µl of 6 M urea/200 mM NH_4_HCO_3_ was added to the beads. The samples were then reduced with DTT (30 nmol, 37 °C, 60 min), alkylated in the dark with iodoacetamide (60 nmol, 25 °C, 30 min) and diluted to 1 M urea/200 mM NH_4_HCO_3_ for trypsin digestion (1 µg, 37 °C, 8 h: Promega cat # V5113). To identify the RNF169 phosphorylation sites, bacterially expressed and purified RNF169 was incubated for 20 min at 30 °C with purified GST-DYRK1A in phosphorylation buffer. Two biological replicas were loaded onto a SDS gel prior to in-gel digestion with trypsin. The peptide mixes were then acidified with formic acid and desalted using Ultra Micro Spin Columns C18 (The Nest Group Inc) prior to LC-MS/MS analysis. Samples were analyzed on a LTQ-Orbitrap Velos Pro mass spectrometer (Thermo Fisher Scientific) coupled to an EASY-nLC 1000 LC system (Thermo Fisher Scientific). Details on the MS experiments and on peptide and protein identification are provided in Supplementary Methods).

### Interactome computational analysis

Keratins, immunoglobulins and trypsin were eliminated from the datasets and in addition, proteins with less than 4 identified spectra in the bait samples were removed from the downstream analysis. The SAINT Express algorithm was used to identify DYRK1A interactors^27^. The proteins identified were analyzed by SAINT-Express comparing: i) the number of SpCs from the specific DYRK1A immunoprecipitation relative to those found in the mock immunoprecipitation (using mouse or rabbit IgGs depending on the DYRK1A Ab); or ii) the number of SpCs in the HeLa WT cells *vs* HeLa-DYRK1A^KO^ cells obtained using the specific DYRK1A Abs. The SAINT score produced by the software (SS) was used for further analysis of the data. Interactors were classified as follows: “high” confidence interactor, SS > 0.6; “low” confidence interactor, 0.6 ≤ SS > 0.4; “non-specific” interactors, SS ≤ 0.4. Given that DYRK1A is a kinase and that transient interactions are to be expected, we considered all proteins detected in at least one experiment for each Ab.

For further comparison of the results obtained with the different Abs, a pipeline was implemented to take into account the efficiency of DYRK1A enrichment in each of the experiments and the molecular weight of the interactors. An enrichment score (ES) was obtained: (i) correcting the enrichment of each prey for non-specific binding to the Ab by subtracting the sum of the SpCs identified in the mock purification (control IgG or HeLa-DYRK1A^KO^) from the sum of the SpCs identified in the DYRK1A-immunoprecipitates; (ii) the filtered cumulative SpC values were normalized to the molecular weight of the preys to provide a measure of relative protein abundance in the immunocomplexes; and (iii) the values were normalized to the specific enrichment of the bait in each purification to enable comparisons to be made between experiments. The computational tools used for the different analysis are listed in the Supplementary Methods.

### Statistical analysis

Statistical analyses were performed using the GraphPad Prism v6.0c (GraphPad Software), which was also used to generate the bar graphs and boxplots. The data in the graphs represents the mean ± standard error of the mean (SEM) of the independent experiments or standard deviation (SD) for technical replicates. Tukey’s method was used to plot the whiskers and outliers in the boxplots. Samples were evaluated for normality using a Shapiro-Wilk test. Statistically significant differences in pairwise comparisons were defined using a two-tailed, unpaired Student’s *t*-test for normal samples, and with a two-tailed, unpaired Mann-Whitney U test for samples not passing the test of normality. For experiments in which the control situation was assigned a value of 100, a one-sample Student’s *t*-test was used. A P value ≤ 0.05 was considered significant.

## Supplementary information

Supplementary Data

Dataset 1

Dataset 2

Dataset 3

## Data Availability

The raw proteomics data and the analysis of the results (peptide and protein identification) have been deposited at the PRIDE repository (https://www.ebi.ac.uk/pride/archive) with the data set identifier PXD011925.
