# Supplementary material for: A comprehensive proteomics-based interaction screen that links DYRK1A to RNF169 and to the DNA damage response
Source: Sci Rep. 2019 Apr 12;9:6014. doi: 10.1038/s41598-019-42445-x (PMC6461666; doi:10.1038/s41598-019-42445-x)
Supplement: Supplementary file 1 — Supplementary Data [file 41598_2019_42445_MOESM1_ESM.pdf]

A comprehensive proteomics-based interaction screen that links DYRK1A to RNF169 and to the DNA damage response

Julia Roewenstrunk, Chiara Di Vona, Jie Chen, Eva Borras, Chao Dong, Krisztina Arató, Eduard Sabidó, Michael S.Y. Huen and Susana de la Luna

Supplementary Data

Supplementary Methods

Supplementary Figure S1, related to Figure 1

Supplementary Figure S2, related to Figure 2

Supplementary Figure S3, related to Figure 3

Supplementary Figure S4, related to Figure 4

Supplementary Figure S5, related to Figure 5

Supplementary Figure S6, related to Figure 5

Supplementary Figure S7, related to Figure 6

Supplementary Figure S8, related to Figure 7

Supplementary Dataset 1, AP-MS analysis of experiments with anti-DYRK1A antibodies *vs* IgG control

Supplementary Dataset 2, AP-MS analysis of experiments with anti-DYRK1A antibodies in HeLa cells *vs* HeLa *DYRK1A*^{KO}

Supplementary Dataset 3, DYRK1A interactors/substrates

Supplementary Methods

Plasmids

The plasmids used to express human DYRK1A (754 aa isoform: Accession number NM_130436) tagged with an N-terminal influenza hemagglutinin (HA) or a green fluorescent protein (GFP), and those with the corresponding kinase-dead version of DYRK1A (K179R), have been already described[1]. Here, a FLAG-tagged version was also generated (763 aa isoform: NM_130435). Plasmids to express DYRK1A internal deletion mutants Δ93-102, Δ97-113, Δ79-113, Δ114-123 were generated by site-directed mutagenesis (QuickChange Site-directed Mutagenesis Kit, Stratagene) of pHA-DYRK1A (Table S1 for the sequence of the mutagenic oligonucleotides). The open reading frames (ORF) of the human DYRK1B (629 aa isoform; NM_004714), DYRK2 (601 aa isoform; NM_006482), DYRK3 (588 aa isoform; NM_003582) were fused to an N-terminal HA tag in pcDNA3 (Invitrogen). The vector to express human DYRK4 (520 aa isoform; NM_003845) has already been described elsewhere[2]. Plasmids to express human RNF168 or RNF169 and their deletion mutants have been described previously[3,4]. To generate a RNF169 catalytically inactive protein (C68S) or mutants of the DYRK1A phosphosites, site-directed mutagenesis was performed with specific primers (Table S1). To express RNF169 protein fragments in bacteria as glutathione-S transferase (GST) fusion proteins (Table S2), synthetic DNA sequences (GeneArt™, Thermo Fisher Scientific) were cloned into pGEX-6P1 (Amersham Biosciences). The plasmids generated by site-directed mutagenesis were verified by DNA sequencing.

Generation of the DYRK1A-KO HeLa cell lines

The genetic inactivation of DYRK1A in HeLa cells was achieved through a CRISPR/Cas9 approach. The genomic region of interest (8,717 bp, chr21:38850414-38859130, see Fig. S1E) in the *DYRK1A* gene was deleted by co-

transfecting vectors carrying SpCas9 (BB)-2A-GFP (PX458; Addgene #48138; [5]) and two guide RNAs (gRNA), designed with the assistance of the CRISPR Design Tool (crispr.mit.edu). Two days after transfection, cells were FACS sorted, colonies were expanded individually and their genetic modification was evaluated by PCR analysis of genomic DNA (Fig. S1F). PCR products were sequenced to confirm the status of the deletion. See Table S3 for the list of guide RNA sequences and primers used to confirm the targeted deletion.

Lentiviral infection

For silencing experiments, the following lentivectors (derived from pLKO.1-puro) were obtained from the Sigma Mission collection: shRNA control, non-targeting vector (SHC001); shRNAs to DYRK1A: sh1-TRCN0000022999, sh2-TRCN0000199464, sh3-TRCN0000010613; shRNAs to RNF169: RCN0000265616. Lentiviral particles were generated by calcium/phosphate co-transfection of the following plasmids into HEK-293T cells: pCMVΔR8.91[6], kindly provided by D. Trono, Ëcole Polytechnique Fédérale de Lausanne, Switzerland); pVSV-G (Addgene #8454[7]); and the appropriate shRNA lentivector (Clontech). After 48 h, the culture supernatants were filtered and applied directly to cells for infection, thereafter selecting the cells for 3-5 days with 1.5 µg/ml puromycin.

Preparation of cell lysates

To prepare the total cell lysates, cells were resuspended in SDS-lysis buffer (25 mM Tris-HCl [pH 7.5], 1% sodium dodecyl sulfate [SDS], 1 mM EDTA, 10 mM sodium pyrophosphate, 20 mM ß-glycerolphosphate) and heated for 15 min at 98 ºC. Soluble extracts were prepared by incubating the cells for 20 min at 4 ºC in lysis buffer 1 (50 mM Hepes [pH 7.4], 150 mM NaCl, 2 mM EDTA, 1% Nonidet P-40 [NP-40]) or RIPA buffer (50 mM Tris-HCl [pH 7.4], 150 mM NaCl, 1 mM EDTA, 1% Triton X-100, 1% sodium deoxycholate, 0.1% SDS), in the presence of a protease

inhibitor cocktail (cOmplete Mini, Roche Diagnostic) and phosphatase inhibitors (2 mM sodium orthovanadate, 30 mM sodium pyrophosphate, 25 mM NaF) for 20 min at 4ºC, followed by centrifugation at 13,000x*g* for 30 min at 4 ºC. Subcellular fractionation of the HeLa cells was carried out with the NE-PER kit (Thermo Scientific), according to the manufacturer's instructions. The cell pellet, corresponding to the insoluble nuclear extract was resuspended in SDS-lysis buffer to recover the nuclear insoluble/chromatin fraction. Protein concentrations were determined with the BCA Protein Assay kit (Pierce), according to the manufacturer's indications.

Western blotting

Cell lysates were resolved on SDS-polyacrylamide gels and the proteins were transferred onto Hybond-ECL nitrocellulose membranes (Amersham Biosciences). The membranes were then blocked for 1 h at room temperature with 10% skimmed milk (Sigma) or 6% BSA (Calbiochem) diluted in TBS-T (10 mM Tris-HCl [pH 7.5], 100 mM NaCl, 0.1% Tween-20), and then probed for 8 h at 4 ºC with the primary Abs. The membranes were washed with TBS-T and then incubated with horseradish peroxidase (HRP)-conjugated secondary antibodies for 1 h at room temperature. Primary and secondary antibodies (Table S4) were diluted in 5% skimmed milk or 3% BSA (phosphoantibodies) in TBS-T. After three washes for 10 min with TBS-T, the membranes were revealed using Western Lightning® Plus ECL (Perkin Elmer) and examined in a LAS-3000 image analyzer (Fuji PhotoFilm, Tokyo, Japan) with the LAS3000-pro software. The signals in Western blots (WB) were quantified using the image software Fiji[8].

Purification of fusion proteins expressed in bacteria

To produce the GST-fusion proteins, GST-fusion constructs were transformed into *Escherichia coli* BL21 (DE3) pLysS B F' cells (Stratagene) and the production of the

fusion proteins was induced by adding 0.1 mM isopropyl ß-D-1-thiogalactopyranoside for 3 h at 37 ºC. To purify the GST-fusion proteins, the bacterial cultures were centrifuged for 15 min at 6,000xg at 4 ºC and the cell pellets were resuspended in GST-lysis buffer (10 mM Tris-HCl [pH 8], 100 mM NaCl, 0.5% NP-40, 1 mM EDTA) containing a protease inhibitor cocktail (cOmplete Mini, Roche Diagnostic). After sonication (3 pulses for 15 sec and 10% amplitude in a Branson Sonifier-250), the samples were centrifuged (15 min/10,000xg at 4ºC), and the supernatant was incubated for 1.5 h at 4 ºC with 100 µl of glutathione-Sepharose beads equilibrated in GST-lysis buffer (Amersham Biosciences). The GST fusion proteins were used either bound to the beads or after elution with elution buffer (10 mM L-glutathione reduced [Sigma], 50 mM Tris-HCl [pH 8]). In the latter case, before use the proteins were dialyzed against the dialysis buffer (50 mM Hepes [pH 7.4], 150 mM NaCl, 2 mM EDTA) at 4 ºC overnight using dialysis membrane tubing (MWCO 12-14,000, Spectra/Por).

To express the myelin basic protein (MBP)-fusion proteins, the same protocol as that described above was used except that the bacteria were grown in the presence of 0.2% glucose, resuspended in MBP-lysis buffer (20 mM Tris-HCl [pH 7.5], 200 mM NaCl, 0.5% NP-40, 1 mM EDTA, 1 mM DTT) containing a protease inhibitor cocktail, and purified using amylose beads (New England Lab).

Pull-down experiments

The MBP-fusion proteins bound to amylose beads were used as bait and *in vitro* translated DYRK1A protein as prey. The TnT T7 Coupled Reticulocyte System (Promega) was used for *in vitro* translation in the presence of [^{35}S]-methionine (1,000 Ci/mmol, Amersham Biosciences), according to the manufacturer's recommendations. Equal amounts of *in vitro* translated proteins were added to the purified MBP-fusion proteins bound to the beads in MBP buffer and incubated for 1 h rolling at 4 ºC. The samples were washed three times with MBP-wash buffer (20

mM Hepes-KOH, 150 mM KCl, 1% NP-40, 5 mM EDTA) and the proteins were visualized by Coomassie blue staining of SDS-PAGE gels or by exposure of the dried gels to film.

Mass spectrometry analysis

Tryptic peptides were loaded onto the 2 cm Nano Trap column with an inner diameter of 100 μm and packed with 5 μm C18 particles (Thermo Fisher Scientific). The peptides were separated by reverse-phase chromatography on a 25 cm column with an inner diameter of 75 μm and packed with 1.9 μm C18 particles (Nikkyo Technos Co). Chromatographic gradients started at 93% buffer A (0.1% formic acid in water) and 7% buffer B (0.1% formic acid in acetonitrile) with a flow rate of 250 nl/min for 5 min, gradually increasing to 65% buffer A and 35% buffer B over 60 min. After each analysis, the column was washed for 15 min with 10% buffer A and 90% buffer B.

The mass spectrometer was operated in positive ionization mode with the nanospray voltage set at 2.1 kV and source temperature at 300 °C. Ultramark 1621 was used for external calibration of the Fourier Transform mass analyzer prior to analysis, and an internal calibration was performed using the background polysiloxane ion signal at m/z 445.1200. Acquisition was achieved in data-dependent acquisition (DDA) mode and full MS scans with 1 microscan at a resolution of 60,000 were used over a mass range of m/z 350-2000, with detection in the Orbitrap. Auto gain control (AGC) was set to 1E6, dynamic exclusion (60 s) and charge state filtering to disqualify singly charged peptides was activated. In each cycle of DDA analysis and following each survey scan, the twenty most intense ions with multiple charged ions above a threshold ion count of 5,000 were selected for fragmentation. Fragment ion spectra were produced by collision-induced dissociation at a normalized collision energy of 35% and they were acquired in the ion trap mass analyzer. The AGC was set to 1E4, with an isolation

window of 2.0 m/z, an activation time of 10 ms and a maximum injection time of 100 ms, and all the data were acquired with Xcalibur software v2.2. Digested bovine serum albumin (New England Biolabs, #P8108S) was analyzed between each sample to avoid sample carryover and to assure stability of the instrument.

The spectra acquired were analyzed using the Proteome Discoverer software suite (v1.4, Thermo Fisher Scientific) and the Mascot search engine (v2.5, Matrix Science, [9]). The data was searched against a SwissProt Human database with a list of common contaminants and all the corresponding decoy entries. For peptide identification, a precursor ion mass tolerance of 7 ppm was used at the MS1 level, trypsin was chosen as the enzyme and up to three missed cleavages were allowed. The fragment ion mass tolerance was set to 0.5 Da for MS2 spectra. Oxidation of methionine and N-terminal protein acetylation were used as variable modifications, while cysteine carbamidomethylation was set as a fixed modification. For RNF169 kinase experiments, phosphorylation of Ser, Thr and Tyr residues were also considered as variable modifications. The false discovery rate for peptide identification was set to a maximum of 5%.

Computational analysis

The list of reported DYRK1A interactors and substrates was compiled from a literature search of Pubmed and with data from BioGRID[10] (thebiogrid.org). Conversion of Uniprot identifiers to Gene symbol and Gene ID was done with bioDBnet[11] (biodbnet-abcc.ncifcrf.gov/db/db2db.php). Venn diagrams were generated with BioVenn[12] (www.biovenn.nl) and the networks were generated with Cytoscape[13], using information from STRING[14]. Protein abundance in HeLa cells were obtained from the PaxDb database[15] (pax-db.org), and it corresponds to dataset 9606/455 [16]. The CRAPome repository[17] was interrogated online (www.crapome.org). Analysis of gene ontology (GO) and pathway enrichment was performed using the Enrichr[18] (amp.pharm.mssm.edu/Enrichr) and DAVID[19]

(david.ncifcrf.gov) web servers. Scatter plots were generated using R[20] (www.R-project.org). The subcellular localization of the target proteins was extracted from UniProt[21] (www.uniprot.org) and amphipathic helices were predicted with HeliQuest[22] (heliquest.ipmc.cnrs.fr). The public databases and tools of the National Centre for Biotechnology Information (NCBI, www.ncbi.nlm.nih.gov) were used to search and analyze the protein and DNA sequences. Proteins were aligned with the free T-Coffee Multiple Sequence Alignment software[23] using the default settings (tcoffee.crg.cat). Accession numbers for the sequences are provided in Table S5. Phosphosites were searched using the PhosphoSitePlus database[24] (www.phosphosite.org).

References

1 Alvarez, M., Estivill, X. & de la Luna, S. DYRK1A accumulates in splicing speckles through a novel targeting signal and induces speckle disassembly. *J Cell Sci* **116**, 3099-3107 (2003).

2 Papadopoulos, C., Arato, K., Lilienthal, E., Zerweck, J., Schutkowski, M., Chatain, N., Muller-Newen, G., Becker, W. & de la Luna, S. Splice variants of the dual specificity tyrosine phosphorylation-regulated kinase 4 (DYRK4) differ in their subcellular localization and catalytic activity. *J Biol Chem* **286**, 5494-5505 (2011).

3 Chen, J., Feng, W., Jiang, J., Deng, Y. & Huen, M. S. Ring finger protein RNF169 antagonizes the ubiquitin-dependent signaling cascade at sites of DNA damage. *J Biol Chem* **287**, 27715-27722 (2012).

4 An, L., Jiang, Y., Ng, H. H., Man, E. P., Chen, J., Khoo, U. S., Gong, Q. & Huen, M. S. Dual-utility NLS drives RNF169-dependent DNA damage responses. *Proc Natl Acad Sci U S A* **114**, E2872-E2881 (2017).

5 Ran, F. A., Hsu, P. D., Wright, J., Agarwala, V., Scott, D. A. & Zhang, F. Genome engineering using the CRISPR-Cas9 system. *Nat Protoc* **8**, 2281-2308 (2013).

6 Zufferey, R., Nagy, D., Mandel, R. J., Naldini, L. & Trono, D. Multiply attenuated lentiviral vector achieves efficient gene delivery in vivo. *Nat Biotechnol* **15**, 871-875 (1997).

7 Stewart, S. A., Dykxhoorn, D. M., Palliser, D., Mizuno, H., Yu, E. Y., An, D. S., Sabatini, D. M., Chen, I. S., Hahn, W. C., Sharp, P. A., Weinberg, R. A. & Novina, C. D. Lentivirus-delivered stable gene silencing by RNAi in primary cells. *RNA* **9**, 493-501 (2003).

8 Schindelin, J., Arganda-Carreras, I., Frise, E., Kaynig, V., Longair, M., Pietzsch, T., Preibisch, S., Rueden, C., Saalfeld, S., Schmid, B., Tinevez, J. Y., White, D.

J., Hartenstein, V., Eliceiri, K., Tomancak, P. & Cardona, A. Fiji: an open-source platform for biological-image analysis. *Nat Methods* **9**, 676-682 (2012).

9 Perkins, D. N., Pappin, D. J., Creasy, D. M. & Cottrell, J. S. Probability-based protein identification by searching sequence databases using mass spectrometry data. *Electrophoresis* **20**, 3551-3567 (1999).

10 Stark, C., Breitkreutz, B. J., Reguly, T., Boucher, L., Breitkreutz, A. & Tyers, M. BioGRID: a general repository for interaction datasets. *Nucleic Acids Res* **34**, D535-539 (2006).

11 Mudunuri, U., Che, A., Yi, M. & Stephens, R. M. bioDBnet: the biological database network. *Bioinformatics* **25**, 555-556 (2009).

12 Hulsen, T., de Vlieg, J. & Alkema, W. BioVenn - a web application for the comparison and visualization of biological lists using area-proportional Venn diagrams. *BMC Genomics* **9**, 488 (2008).

13 Shannon, P., Markiel, A., Ozier, O., Baliga, N. S., Wang, J. T., Ramage, D., Amin, N., Schwikowski, B. & Ideker, T. Cytoscape: a software environment for integrated models of biomolecular interaction networks. *Genome Res* **13**, 2498-2504 (2003).

14 Szklarczyk, D., Morris, J. H., Cook, H., Kuhn, M., Wyder, S., Simonovic, M., Santos, A., Doncheva, N. T., Roth, A., Bork, P., Jensen, L. J. & von Mering, C. The STRING database in 2017: quality-controlled protein-protein association networks, made broadly accessible. *Nucleic Acids Res* **45**, D362-D368 (2017).

15 Wang, M., Herrmann, C. J., Simonovic, M., Szklarczyk, D. & von Mering, C. Version 4.0 of PaxDb: Protein abundance data, integrated across model organisms, tissues, and cell-lines. *Proteomics* **15**, 3163-3168 (2015).

16 Nagaraj, N., Wisniewski, J. R., Geiger, T., Cox, J., Kircher, M., Kelso, J., Paabo, S. & Mann, M. Deep proteome and transcriptome mapping of a human cancer cell line. *Mol Syst Biol* **7**, 548 (2011).

17 Mellacheruvu, D., Wright, Z., Couzens, A. L., Lambert, J. P., St-Denis, N. A., Li, T., Miteva, Y. V., Hauri, S., Sardiu, M. E., Low, T. Y., Halim, V. A., Bagshaw, R. D., Hubner, N. C., Al-Hakim, A., Bouchard, A., Faubert, D., Fermin, D., Dunham, W. H., Goudreault, M., Lin, Z. Y., Badillo, B. G., Pawson, T., Durocher, D., Coulombe, B., Aebersold, R., Superti-Furga, G., Colinge, J., Heck, A. J., Choi, H., Gstaiger, M., Mohammed, S., Cristea, I. M., Bennett, K. L., Washburn, M. P., Raught, B., Ewing, R. M., Gingras, A. C. & Nesvizhskii, A. I. The CRAPome: a contaminant repository for affinity purification-mass spectrometry data. *Nat Methods* **10**, 730-736 (2013).

18 Kuleshov, M. V., Jones, M. R., Rouillard, A. D., Fernandez, N. F., Duan, Q., Wang, Z., Koplev, S., Jenkins, S. L., Jagodnik, K. M., Lachmann, A., McDermott, M. G., Monteiro, C. D., Gundersen, G. W. & Ma'ayan, A. Enrichr: a comprehensive gene set enrichment analysis web server 2016 update. *Nucleic Acids Res* **44**, W90-97 (2016).

19 Huang da, W., Sherman, B. T. & Lempicki, R. A. Systematic and integrative analysis of large gene lists using DAVID bioinformatics resources. *Nat Protoc* **4**, 44-57 (2009).

20 Team, R. C. R: A language and environment for statistical computing. *R Foundation for Statistical Computing, Vienna, Austria.*, (2015).

21 UniProt Consortium, T. UniProt: the universal protein knowledgebase. *Nucleic Acids Res* **46**, 2699 (2018).

22 Gautier, R., Douguet, D., Antonny, B. & Drin, G. HELIQUEST: a web server to screen sequences with specific alpha-helical properties. *Bioinformatics* **24**, 2101-2102 (2008).

23 Di Tommaso, P., Moretti, S., Xenarios, I., Orobitg, M., Montanyola, A., Chang, J. M., Taly, J. F. & Notredame, C. T-Coffee: a web server for the multiple sequence alignment of protein and RNA sequences using structural information and homology extension. *Nucleic Acids Res* **39**, W13-17 (2011).

24 Hornbeck, P. V., Zhang, B., Murray, B., Kornhauser, J. M., Latham, V. & Skrzypek, E. PhosphoSitePlus, 2014: mutations, PTMs and recalibrations. *Nucleic Acids Res* **43**, D512-520 (2015).

25 Alvarez, M., Altafaj, X., Aranda, S. & de la Luna, S. DYRK1A autophosphorylation on serine residue 520 modulates its kinase activity via 14-3-3 binding. *Mol Biol Cell* **18**, 1167-1178 (2007).

Table S1: Oligonucleotides for site-directed mutagenesis.

Name	Oligonucleotide (5'->3')
DYRK1AΔ93-102	CTCATTAATATGCTTGTATGTGGGAGCAGTTGCTGGGT
DYRK1AΔ97-113	GGTGTCTTCGCTTCTTTTTAAGTTTTCTCAGGGGAGCAG
DYRK1AΔ79-113	GCAACCTCTAACTAACCAGAAAAAGAAGCGAAGACACC
DYRK1AΔ114-123	GAGGTTTACTATGCAGGAGACGATTCTAGTCATAAG
RNF169-C68S	GCCGGAGGAATCGGGC**AGC**GCCGGGTGCCTGGAGC
RNF169-S688A	ACAATGAGAGGCGGACTGTG**GCC**CGGCGAAAAGGAAG
RNF169-S688D	ACAATGAGAGGCGGACTGTG**GAC**CGGCGAAAAGGAAG
RNF169-S368A	CATAAGCCAGAGCGTTCTGTC**GCC**CCTGAGAGCAATGA
RNF169-S403A	TCCCTGATGGCCGTGT**GCT**AGCTCCTCTCATCATCAAA
RNF169-S12A	GAGTACTCGGGCCTCT**GCC**GCGGCGGCAGC

Table S2: RNF169 fragments expressed in bacteria as N-terminal GST fusion proteins.

Name	Size
RNF169N	5 - 65
RNF169N-S12A	5 - 65, with Ser12 mutated to Ala
RNF169N-S12/20A	5 - 65, with Ser12 and Ser20 mutated to Ala
RNF169M	330 - 430
RNF169M-S339A	330 - 430, with Ser339 mutated to Ala
RNF169M-S368A	330 - 430, with Ser368 mutated to Ala
RNF169M-S403A	330 - 430, with Ser403 mutated to Ala
RNF169M-S339/403A	330 - 430, with Ser339 and Ser403 mutated to Ala
RNF169M-S339/368/403A	330 - 430, with Ser339, Ser368 and Ser403 mutated to Ala
RNF169C	658 - 708
RNF169C-T686A	658 - 708, with Thr686 mutated to Ala
RNF169C-S688A	658 - 708, with Ser688 mutated to Ala
RNF169C-S693A	658 - 708, with Ser693 mutated to Ala
RNF169C-T686/S688/693A	658 - 708, with Thr686, Ser688 and Ser693 mutated to Ala

Table S3: Oligonucleotides for the CRISPR/Cas9 experiments.

Name	Oligonucleotide (5'->3')
gRNA1 (50-54)	ACATGCAGGTTACAGAAGA
gRNA2 (64-41)	TACATGGTATGCTACATGGA
PCR1f-WT	GGCATATGATCGTGTGGAGC
PCR1r-WT	ATGCTGCCAAGCTGATGAAGA
PCR2f-KO	AGGAGCAGTTGGAGGGAATG

Table S4: Properties and working dilutions of the antibodies used in the study.

Primary antibody	Host	Working dilution	Source
Anti-53BP1	Rabbit	IF (1:2000)	Novus Biological (NB100-304)
Anti-DCAF7	Rabbit	WB (1:1000)	Sigma (HPA022948)
Anti-DYRK1A-C1	Mouse	WB (1:1000)<br>IP (3 µg)	Abnova (H00001859-M01)
Anti-DYRK1A-C2	Rabbit	IP (3 µg)	Abcam (ab69811)
Anti-DYRK1A-N	Rabbit	IP (3 µg)	Sigma (D1694)
Anti-FLAG	Mouse	WB (1:5000)<br>IP (2 µg)<br>IF (1:1000)	Sigma (F1804, Clone M2)
Anti-FLAG	Rabbit	IF (1:400)	Cell Signaling (2368)
Anti-GFP	Mouse	WB (1:10000)	Clontech (632380)
Anti-GFP	Rabbit	IF (1:500)	Thermo Fisher (A6455)
Anti-HA	Mouse	WB (1:1000)<br>IP (1 µg)<br>IF (1:1000)	BioLegend (901501)
Anti-H2AX (pS139)	Mouse	IF (1:500)	Millipore (05-636)
Anti-Histone H3	Rabbit	WB (1:5000)	Abcam (ab1791)
Anti-Lamin B1	Goat	WB (1:500)	Santa Cruz (sc-6216)
Anti-RNF168	Rabbit	WB (1:500)	Millipore (ABE367)
Anti-RNF169	Rabbit	WB (1:2000)<br>IP (3 µg)	Bethyl Laboratories (A304-097A)
Anti-αTubulin	Mouse	WB (1:50000)	Sigma (T6199)
Anti-Vinculin	Mouse	WB (1:50000)	Sigma (V9131)

Anti-Mouse-HRP	Rabbit	1:2000-10000	Dako (P0260)
Anti-Rabbit-HRP	Goat	1:2000-10000	Dako (P0448)
Anti-Goat-HRP	Rabbit	1:2000-10000	Abcam (ab6741)
Anti-Rabbit-DyLight550	Goat	IF (1:2500)	Bethyl Laboratories (A120-201D3)
Anti-Rabbit-DyLight488	Donkey	IF (1:2500)	Bethyl Laboratories (A120-108D2)
Anti-Mouse-DyLight488	Goat	IF (1:2500)	Bethyl Laboratories (A90-116D3)
Anti-Mouse-AlexaFluor488	Donkey	IF (1:2500)	Invitrogen (A21202)

IF, immunofluorescence; IP, immunoprecipitation; WB, Western blot

Table S5: Accession numbers for the protein sequences used in this work

Name	Species	Accession number
DYRK1A	*Homo sapiens*	NP_569120
DYRK1A	*Xenopus laevis*	NP_001156669
DYRK1 (minibrain)	*Drosophila melanogaster*	NP_728107
MBK1	*Caenorhabditis elegans*	NP_510460
DYRK1B	*Homo sapiens*	NP_004705
RNF169	*Homo sapiens*	NP_001092108
RNF169	*Mus musculus*	NP_780597
RNF169	*Monodelphis domestica*	XP_007491043.1
RNF169	*Xenopus laevis*	XP_018101122.1
RNF169	*Danio rerio*	XP_001336012.2
RNF168	*Homo sapiens*	NP_689830

Supplemental Figures

Supplemental Figure S1, related to Figure 1

(A) Distribution of DYRK1A and DCAF7 in the cytoplasmic/membrane (C), nuclear (N) and chromatin (Ch) fractions of HeLa cells, and the distribution of tubulin, lamin B1 and histone H3 as controls for the different fractions.

(B) Validation of the antibodies used in the AP-MS experiments. The position of the epitopes used to raise the antibodies is shown in a schematic representation of human DYRK1A protein: Ab-N, rabbit polyclonal Ab from Sigma; Ab-C1, mouse monoclonal Ab from Abnova; and Ab-C2, rabbit polyclonal Ab from Abcam. Each of the three Abs or a rabbit IgG (IgG Control) were used in immunoprecipitations of total soluble HeLa cell extracts. Both the lysate and the immunocomplexes were analyzed in WBs probed with DYRK1A Ab-C1.

(C) The kinase activity of DYRK1A-immunocomplexes obtained from HeLa soluble cell extracts with the three specific DYRK1A Abs was assessed using DYRKtide as the substrate, in the presence or absence of the DYRK inhibitor harmine (10 μM). Immunocomplexes obtained with mouse IgGs were used as controls (Control) and the graph shows the mean ± SD of quantifications in triplicate.

(D) Quantification of DYRK1A kinase activity associated to the different antibodies in which the activity associated to Ab-C1 was set as 100 (mean ± SEM; n=5 independent experiments; one-sample Student's *t*-test).

(E) Schematic representation of the genomic structure of human DYRK1A: the boxes represent exons (in red, exons encoding for the catalytic domain) and the lines represent introns. The gene size is indicated. The region deleted by CRISPR/Cas9 is amplified, in which the blue arrows indicate the position of the single-guide RNAs used (gRNA1 and gRNA2) and the red arrows the position of the primers used to confirm the deletion (see Table S3).

(F) PCR Analysis of the genomic DNA from either WT or DYRK1A^{KO} HeLa clones. The PCR products showing the WT and the mutant allele are indicated, together with the primers used and the size of the PCR product detected.

(G) The presence of DYRK1A was analyzed in WBs of total cell extracts from either HeLa WT or HeLa-DYRK1A^{KO} cells. Increasing amounts of the extracts were loaded and tubulin was used as loading control.

(H) DYRK1A immunoprecipitated from HeLa WT or HeLa-DYRK1A^{KO} soluble extracts using the antibodies indicated were analyzed in WBs probed with Ab-C1. Three replicates (r) per condition were performed. The image corresponds to one of the AP-MS experiments.

Supplemental Figure S2, related to Figure 2

(A) Examples of proteins identified in different AP-MS experiments in which either IgGs or HeLa-DYRK1A^{KO} cells were used as controls. The SpCs of each triplicate are shown.

(B) Venn diagram showing the subcellular localization of the proteins in Fig. 2A that are annotated in UniProt.

(C) Proteins in the DYRK1A network shown in Fig. 2B that are associated with GSK3 (according to STRING and the KEA database at Enrichr). Assessment of the GSK3 substrates (circled in red) was carried out by searching PubMed.

Supplemental Figure S3, related to Figure 3

(A) Relative enrichment of DCAF7 in total extracts cell (light orange) and nuclear extracts (dark orange) of HeLa cells in the DYRK1A interactome analysis. The graph shows the enrichment score (ES) for each Ab and cellular compartment (mean ± SEM of two independent experiments).

(B) Co-immunoprecipitation experiments with an anti-FLAG Ab on extracts from HEK-293T cells expressing the proteins indicated. The presence of the expressed

proteins was evaluated in WBs probed with anti-FLAG or anti-HA antibodies. The scheme shows the primary structures of RNF168 and RNF169.

(C) DYRK1A and RNF169 interact directly. A MBP pull-down assay was performed on equal amounts of *in vitro* produced ^{35}S-Met labeled DYRK1A or Luciferase (negative control) as prey, and the unfused MBP or MBP-RNF169 as bait. The samples were fractionated by SDS-PAGE and analyzed by Coomassie blue staining to check for equal loading, or by autoradiography to assess the presence of the prey.

Supplemental Figure S4, related to Figure 4

(A) Identification of the DYRK1A-binding domain in RNF169. HEK-293T cells transiently transfected with the indicated RNF169 expression constructs were lysed and co-immunoprecipitation experiments were performed using anti-HA antibodies. Co-immunoprecipitated proteins were analyzed in WBs probed with anti-HA and anti-FLAG antibodies. Schematic representations of the RNF169 primary sequence and its deletion mutants are shown.

(B) Representation of the RNF169 interacting region in DYRK1A (P92-K102) as an amphipathic helix, as predicted by HeliQuest.

(C) Sequence alignment of the N-terminal region of several DYRK proteins: *Homo sapiens* DYRK1A (Hs, 763 aa isoform), the close paralog DYRK1B and the DYRK1A orthologs in *Xenopus laevis* (Xl), *Drosophila melanogaster* (Dm) and *Caenorhabditis elegans* (Ce). The numbers in red indicate the position of the deletions in the HA-DYRK1A mutant proteins used in Fig. 4C.

(D) Schematic representation of the structure of the human DYRK kinases and their phylogenetic relationship.

(E) Soluble extracts from HEK-293T cells expressing the proteins indicated were immunoprecipitated with anti-FLAG Ab, and both the lysates and the immunocomplexes were analyzed in WBs probed with anti-FLAG and anti-HA.

Supplemental Figure S5, related to Figure 5

(A) Soluble extracts from HEK-293T cells expressing DYRK1A-WT, DYRK1A-KR (kinase inactive) and the DYRK1A deletion mutants indicated were immunoprecipitated with anti-HA and used in IVK assays, in the presence of radioactive labeled ATP and DYRKtide as the substrate. The kinase activity was corrected to the amount of DYRK1A immunoprecipitated. The values for the DYRK1A-WT were arbitrarily set as 1. No statistically significant differences were found when the activity of the DYRK1A mutants was compared to that of the WT protein (n=6-7; one-sample *t*-test). The ability of the DYRK1A proteins to interact with RNF169 is indicated.

(B) A representative IVK experiment is shown to illustrate mutant DYRK1A autophosphorylation.

(C) DYRK1A protein levels were assessed in WBs of HeLa control cells or cells depleted of RNF169 by lentiviral transduction of shRNA. RNF169 depletion was assessed in WBs of total lysates and in RNF169-immunoprecipitates (IP). Vinculin was used as a loading control.

(D) DYRK1A protein was measured in HeLa cells under conditions of RNF169 depletion. The endogenous levels of DYRK1A in control cells (corrected for the loading control) were set as 100 (mean ± SEM, n=5, one-sample Student's *t*-test).

(E) RNF169 phosphorylation in siControl and siDYRK1A transfected HEK-293T cells, corresponding to the representative experiment shown in Fig. 5A. The graph shows the mean ± SEM of two independent experiments (the autoradiography signal corrected to the WB signal), in which the value for the siControl was set as 100 (one-sample Student's *t*-test).

(F) HeLa cells or HeLa-DYRK1A^{KO} cells were transfected to express Flag-tagged RNF169 wild-type (WT) or a version with S12, S368, S403 and S688 mutated to alanine (Mut). The proteins were immunoprecipitated with a Flag antibody and

subjected to a radioactive IVK assay. Phosphorylation was assessed by autoradiography, and the presence of RNF169 in the immunoprecipitates by and expression level of DYRK1A in the lysates was evaluated by Western blot.

Supplemental Figure S6, related to Figure 5

(A) Schematic representation of the RNF169 wild type (WT) and deletion mutants (Δ1, ΔC, ΔN) used in the IVK assays in B. The amino acid numbers for each deletion are indicated and the phosphosites are shown in red.

(B) Soluble extracts from HEK-293T cells transiently expressing the proteins indicated were immunoprecipitated with anti-HA Ab and assessed in an IVK assay in the presence of radioactively labeled ^{32}P-ATP. Samples were fractionated by SDS-PAGE and analyzed by autoradiography. The presence of the proteins in the immunocomplexes was assessed in WBs probed with the anti-HA Ab. The autophosphorylation of DYRK1A and the lack of signal for the kinase-inactive version are considered as internal controls.

(C) Schematic representation of the RNF169 regions used to validate the phosphosites identified by MS (in red).

(D-F) The GST-RNF169 fusion proteins shown in C and the mutants indicated were produced in bacteria, and used in IVK assays in the presence or absence of GST-DYRK1A. The samples were analyzed by autoradiography to assess their phosphorylation and by Coomassie blue staining to check for equal loading. The dotted line in D indicates that the last lane comes from another part of the gel. The asterisk in F indicates a protein band that co-purifies with GST-RNF169C. The value of the autoradiography signal corrected to the Coomassie staining signal was calculated, and that for the WT protein set as 1. The values are shown at the bottom of the images.

(D) RNF169N: a small decrease in the intensity of the signal was observed in the S12A mutant suggesting that this residue is poorly phosphorylated by DYRK1A. A

further small decrease was found in the S12/20A double mutant signal compared to that for S12A; this could suggest that none of the residues are good phosphosites for DYRK1A and that other residues in the fragment could be phosphorylated by DYRK1A *in vitro*.

(E) RNF169M: little changes were detected in the signal for the S339A mutant; however, the intensity of the radioactive signal was reduced in the S368A and S403A mutants, confirming that these two residues are phosphorylated by DYRK1A. Further confirmation came from a similar decrease in intensity in the S339/403A double mutant to that in the single S403A mutant. Finally, no radioactive signal was observed for the triple S339/368/403A mutant. This result would indicate that S368 and S403 account for all the DYRK1A phosphorylation within the central region of RNF169. Moreover, we also conclude that serine residues 352, 409, 417 and threonine 410 are not putative DYRK1A phosphorylation sites.

(F) RNF169C: a doublet was detected at around the same position as GST-RNF169C in the Coomassie stained gel. While the intensity of the lower band was affected by the mutations, the upper band did not, suggesting that it is a bacterial protein co-purifying with GST-RNF169C and that it was phosphorylated by DYRK1A. The signal in the smaller band was weaker in the S688A mutant, while no changes were observed when mutants T686A and S693A were analyzed. A further reduction in signal intensity was found for the triple T686/S688/693A mutant as for the single S688A mutant, suggesting that the phosphorylation of the three sites by DYRK1A might depend on each other. Therefore, S688 in RNF169 is confirmed as a DYRK1A phosphosite, whereas no clear conclusion can be raised for T686 and S693.

Supplemental Figure S7, related to Figure 6

(A) HeLa cells were transiently transfected with the plasmids encoding the proteins indicated and 36 h later, they were subjected to IR (3 Gy, +IR) or left untreated (-

IR). The cells were fixed after a 1 h recovery and immunostained with antibodies against GFP (green) and FLAG (red), counter-staining the nuclei with DAPI (blue). Scale bar, 10 μm.

(B) HEK-293T cells were transiently transfected with the plasmids to express RNF169 or the mutants indicated. Soluble cell extracts were immunoprecipitated with anti-FLAG and the proteins were identified in WBs. Note that there were no differences in the binding of the different RNF169 proteins to endogenous DYRK1A, DCAF7 or the RNF169 interactor USP7 [4].

(C) HeLa cells were irradiated (1 Gy) and allowed to recover for the time indicated, and the DYRK1A immunocomplexes recovered were analyzed in WBs. The autophosphorylation of DYRK1A at S520 [25] was used to assess DYRK1A kinase activity (pS520), with phosphorylation of ataxia telangiectasia mutated (ATM) as a marker of the activation of ATM pathway following IR (pS1981-ATM). Mouse IgG Abs were used as a control (IP Control) and Vinculin as a control of loading.

(D) DYRK1A-associated kinase activity from irradiated HeLa cells assessed in DYRK1A immunoprecipitates and with DYRKtide as a substrate. A representative experiment is shown (mean ± SD of triplicates).

Supplemental Figure S8, related to Figure 7

HeLa cells treated with indicated siRNAs were either irradiated with the indicated doses of IR (A), or processed for Western blotting (B). The graph represents the mean ± SEM from three independent experiments each performed in triplicates.

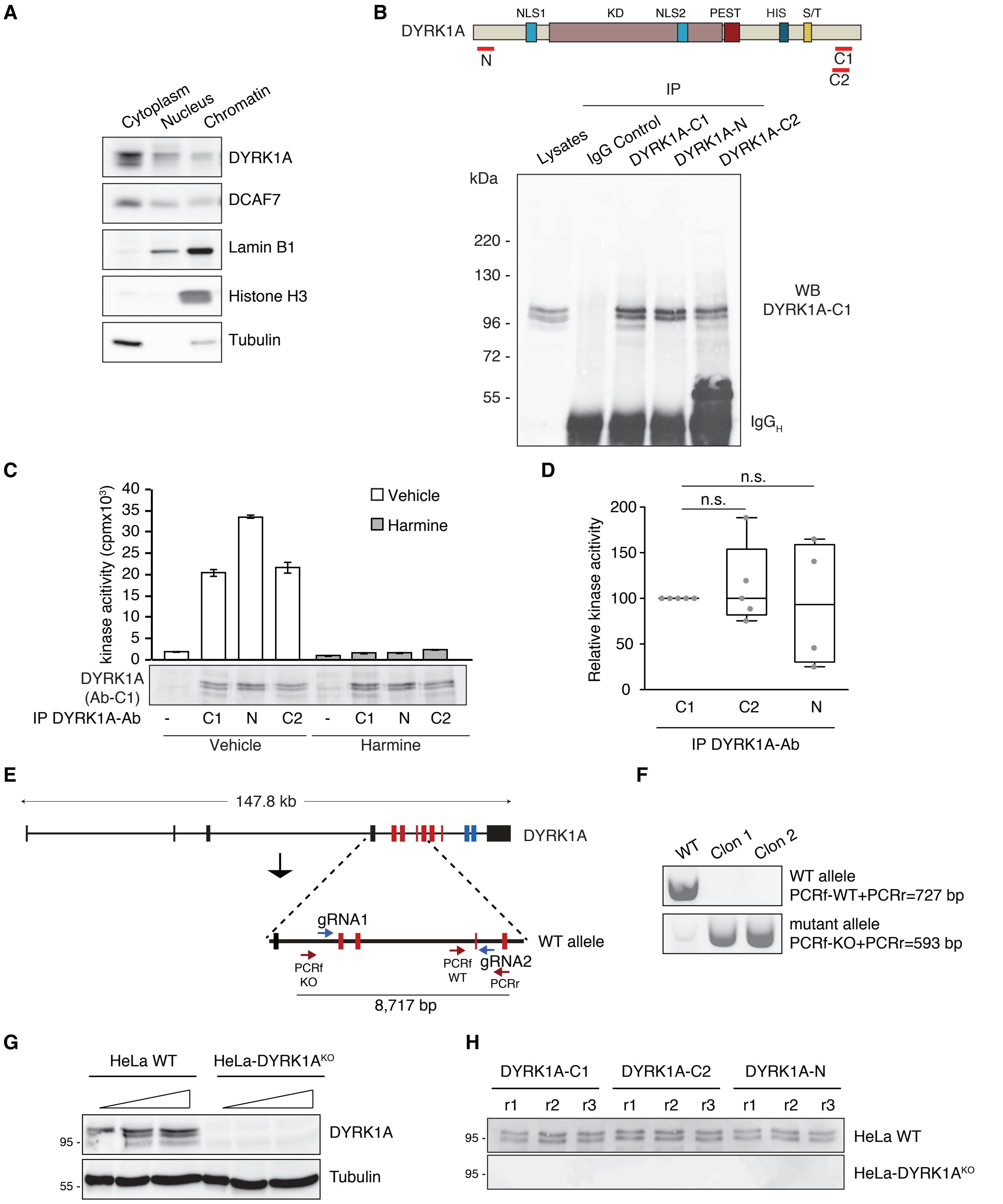
A
Cytoplasm
Nucleus
Chromatin
DYRK1A
DCAF7
Lamin B1
Histone H3
Tubulin
B
DYRK1A
NLS1
KD
NLS2
PEST
HIS
S/T
N
C1
C2
IP
Lysates
IgG Control
DYRK1A-C1
DYRK1A-N
DYRK1A-C2
kDa
220 -
130 -
96 -
72 -
55 -
WB
DYRK1A-C1
IgGH
C
kinase acitivity (cpmx10³)
40
35
30
25
20
15
10
5
0
Vehicle
Harmine
DYRK1A
(Ab-C1)
IP DYRK1A-Ab
- C1 N C2 - C1 N C2
Vehicle
Harmine
D
n.s.
n.s.
Relative kinase activity
200
150
100
50
0
C1
C2
N
IP DYRK1A-Ab
E
147.8 kb
DYRK1A
gRNA1
WT allele
PCRf
KO
PCRf
WT
gRNA2
PCRr
8,717 bp
F
WT
Clon 1
Clon 2
WT allele
PCRf-WT+PCRr=727 bp
mutant allele
PCRf-KO+PCRr=593 bp
G
HeLa WT
HeLa-DYRK1AKO
95 -
55 -
DYRK1A
Tubulin
H
DYRK1A-C1
DYRK1A-C2
DYRK1A-N
r1 r2 r3 r1 r2 r3 r1 r2 r3
95 -
95 -
HeLa WT
HeLa-DYRK1AKO

A

<table>
<tr><th></th><th>Extracts</th><th colspan="2">HeLa^{WT}</th><th>HeLa^{WT}</th><th>HeLa^{KO}</th></tr>
<tr><th></th><th>IP</th><th>DYRK1A</th><th>IgG</th><th>DYRK1A</th><th>DYRK1A</th></tr>
<tr><td rowspan="4">Ab-C1</td><td>DYRK1A</td><td>25|27|30</td><td>0|0|0</td><td>27|24|26</td><td>0|0|0</td></tr>
<tr><td>TRMT61B</td><td>14|13|13</td><td>0|0|0</td><td>22|28|27</td><td>45|40|38</td></tr>
<tr><td>GSPT1</td><td>18|28|22</td><td>0|0|0</td><td>29|34|34</td><td>42|39|40</td></tr>
<tr><td>FGG</td><td>8|9|4</td><td>0|0|0</td><td>4|4|7</td><td>6|5|9</td></tr>
<tr><td rowspan="4">Ab-C2</td><td>DYRK1A</td><td>41|39|35</td><td>0|0|0</td><td>6|8|15</td><td>0|0|0</td></tr>
<tr><td>TRMT61B</td><td>17|16|17</td><td>0|0|0</td><td>7|12|2</td><td>15|15|14</td></tr>
<tr><td>NSUN2</td><td>112|115|109</td><td>0|0|0</td><td>135|146|63</td><td>144|154|98</td></tr>
<tr><td>CPSF1</td><td>9|12|12</td><td>0|0|0</td><td>13|15|20</td><td>31|35|22</td></tr>
<tr><td rowspan="3">Ab-N</td><td>DYRK1A</td><td>36|37|29</td><td>0|0|0</td><td>30|38|28</td><td>0|0|0</td></tr>
<tr><td>CPSF1</td><td>20|20|23</td><td>0|0|0</td><td>2|3|1</td><td>2|2|4</td></tr>
<tr><td>FIP1L1</td><td>14|13|16</td><td>0|0|0</td><td>7|9|10</td><td>7|9|10</td></tr>
</table>

B

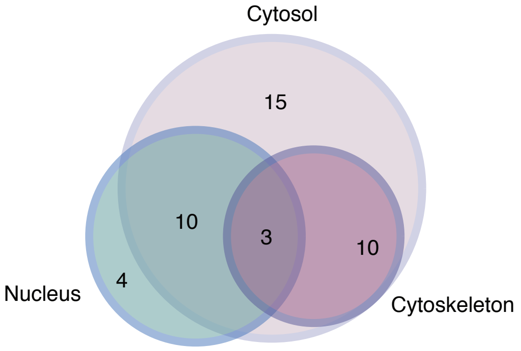

C

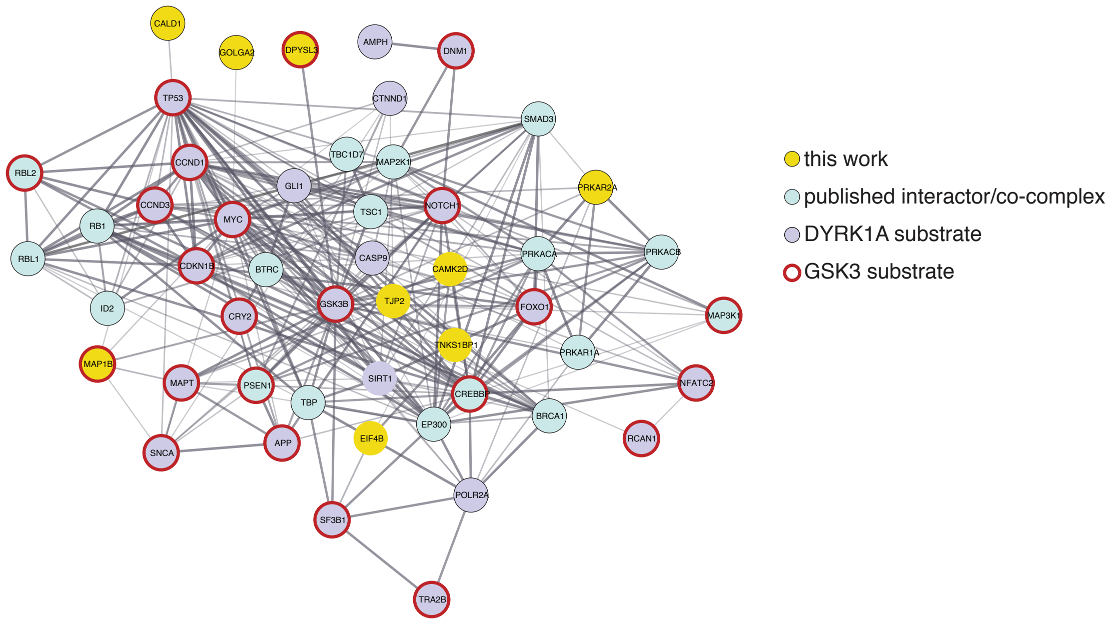

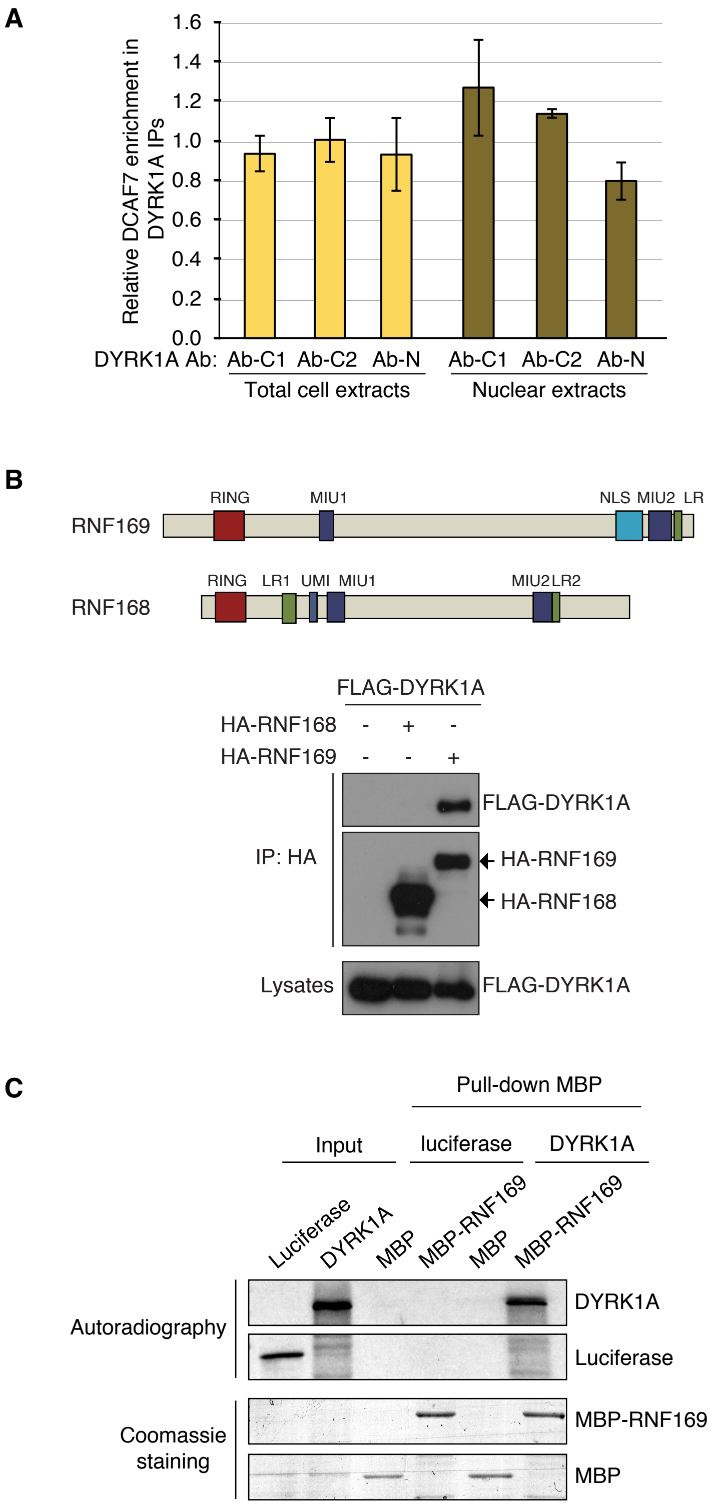
A
Relative DCAF7 enrichment in DYRK1A IPs
1.6
1.4
1.2
1.0
0.8
0.6
0.4
0.2
0.0
DYRK1A Ab: Ab-C1 Ab-C2 Ab-N Ab-C1 Ab-C2 Ab-N
Total cell extracts
Nuclear extracts
B
RNF169
RING
MIU1
NLS MIU2 LR
RNF168
RING LR1 UMI MIU1 MIU2 LR2
FLAG-DYRK1A
HA-RNF168 - + -
HA-RNF169 - - +
IP: HA
FLAG-DYRK1A
HA-RNF169
HA-RNF168
Lysates
FLAG-DYRK1A
C
Pull-down MBP
Input
luciferase
DYRK1A
Luciferase
DYRK1A
MBP
MBP-RNF169
MBP
MBP-RNF169
Autoradiography
DYRK1A
Luciferase
Coomassie staining
MBP-RNF169
MBP

Supplemental Figure S4
Roewenstrunk, Di Vona *et al.*,

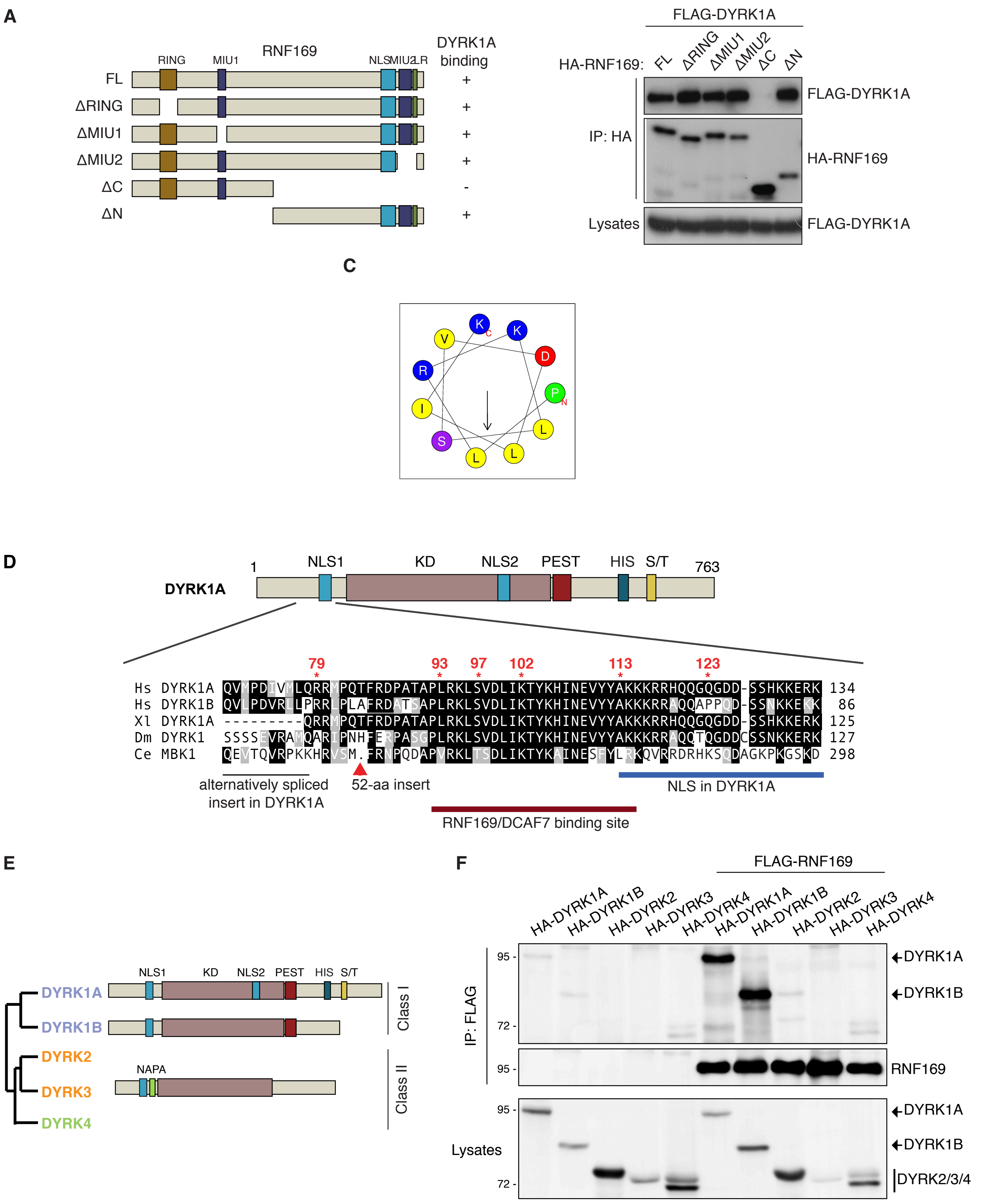

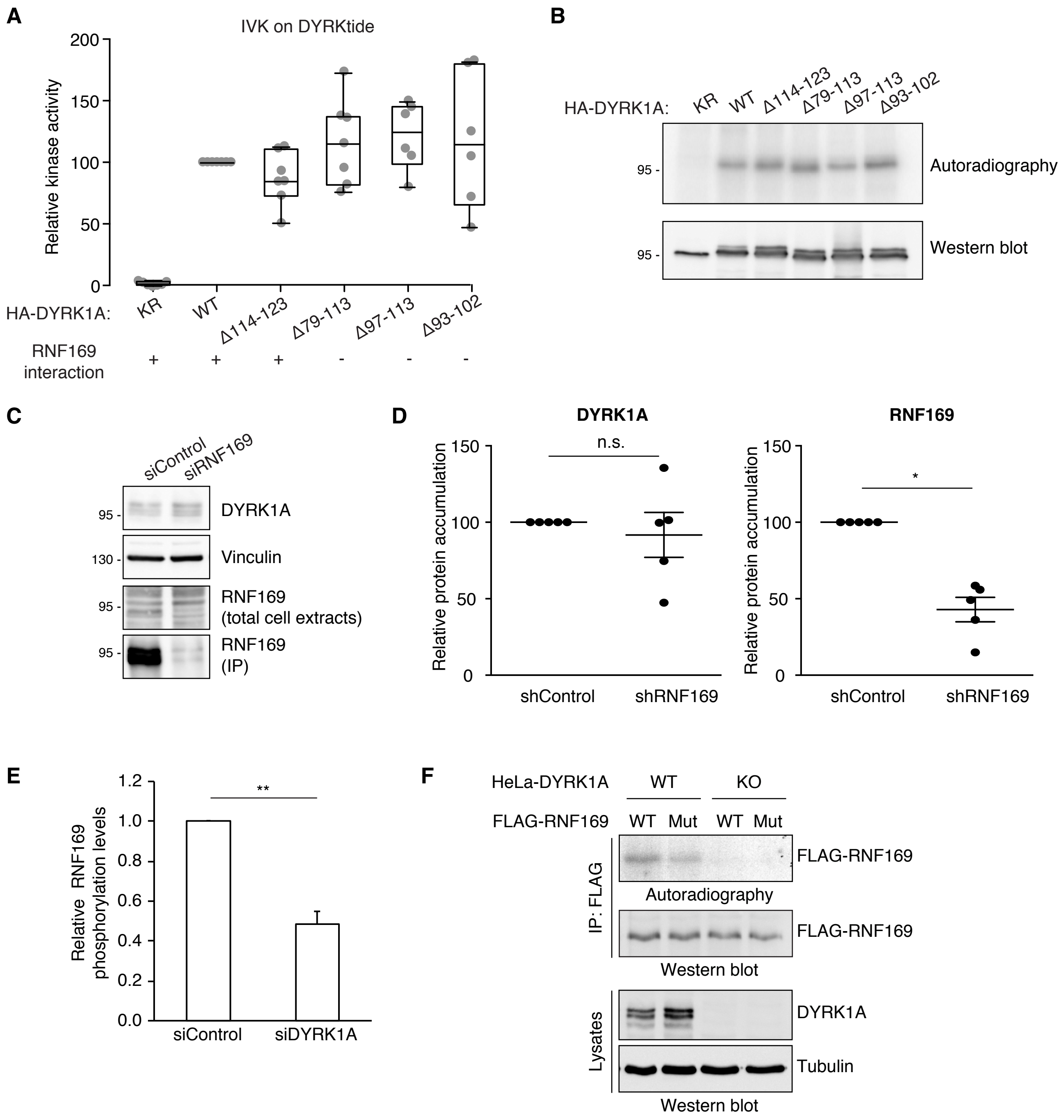

A
IVK on DYRKtide
Relative kinase activity
HA-DYRK1A:
KR
WT
Δ114-123
Δ79-113
Δ97-113
Δ93-102
RNF169 interaction
B
HA-DYRK1A:
Autoradiography
Western blot
C
siControl
siRNF169
DYRK1A
Vinculin
RNF169 (total cell extracts)
RNF169 (IP)
D
DYRK1A
RNF169
Relative protein accumulation
n.s.
shControl
shRNF169
E
Relative RNF169 phosphorylation levels
siControl
siDYRK1A
F
HeLa-DYRK1A
FLAG-RNF169
IP: FLAG
Lysates
FLAG-RNF169
Autoradiography
Western blot
DYRK1A
Tubulin

Supplemental Figure S6
Roewenstrunk, Di Vona *et al.*,

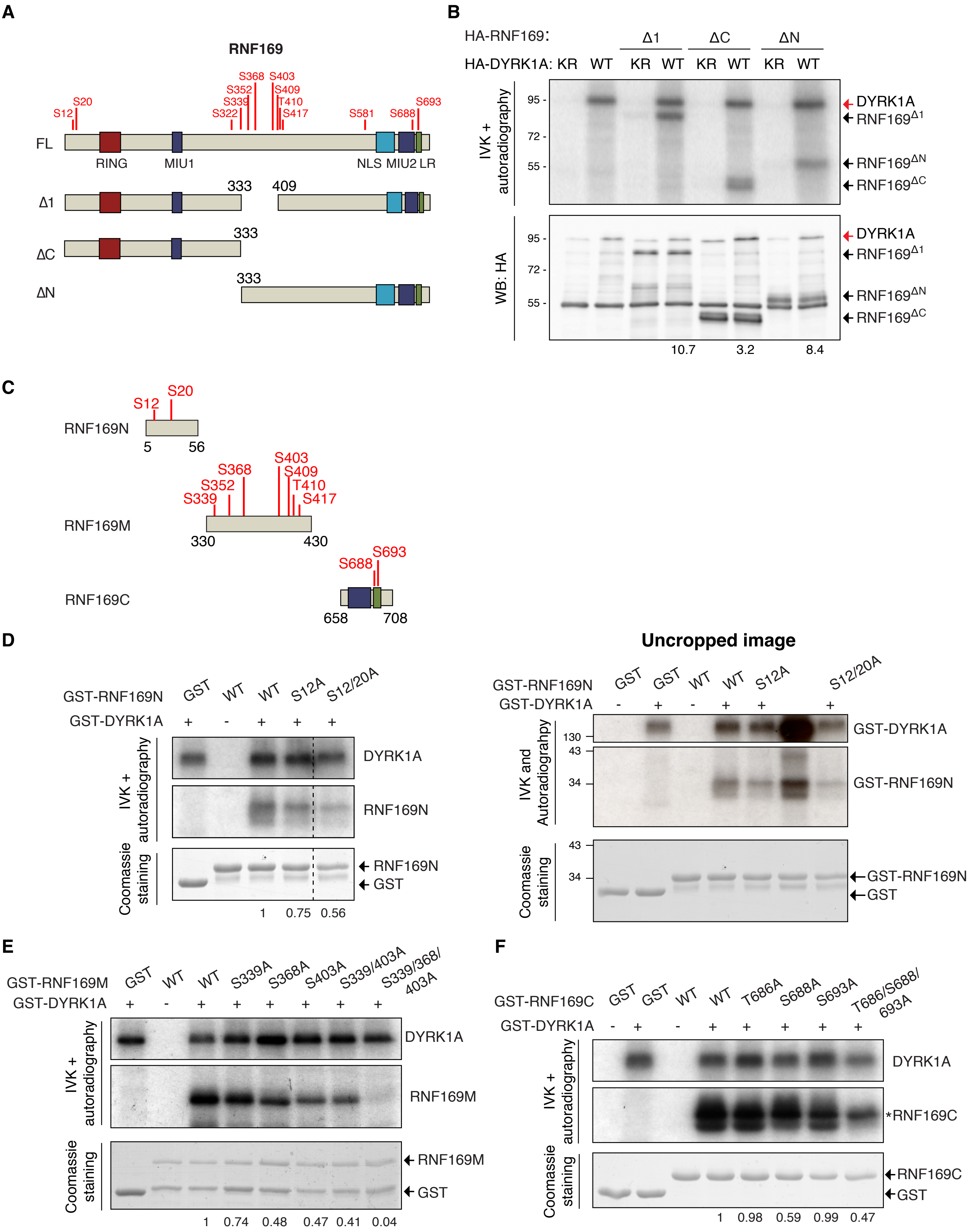

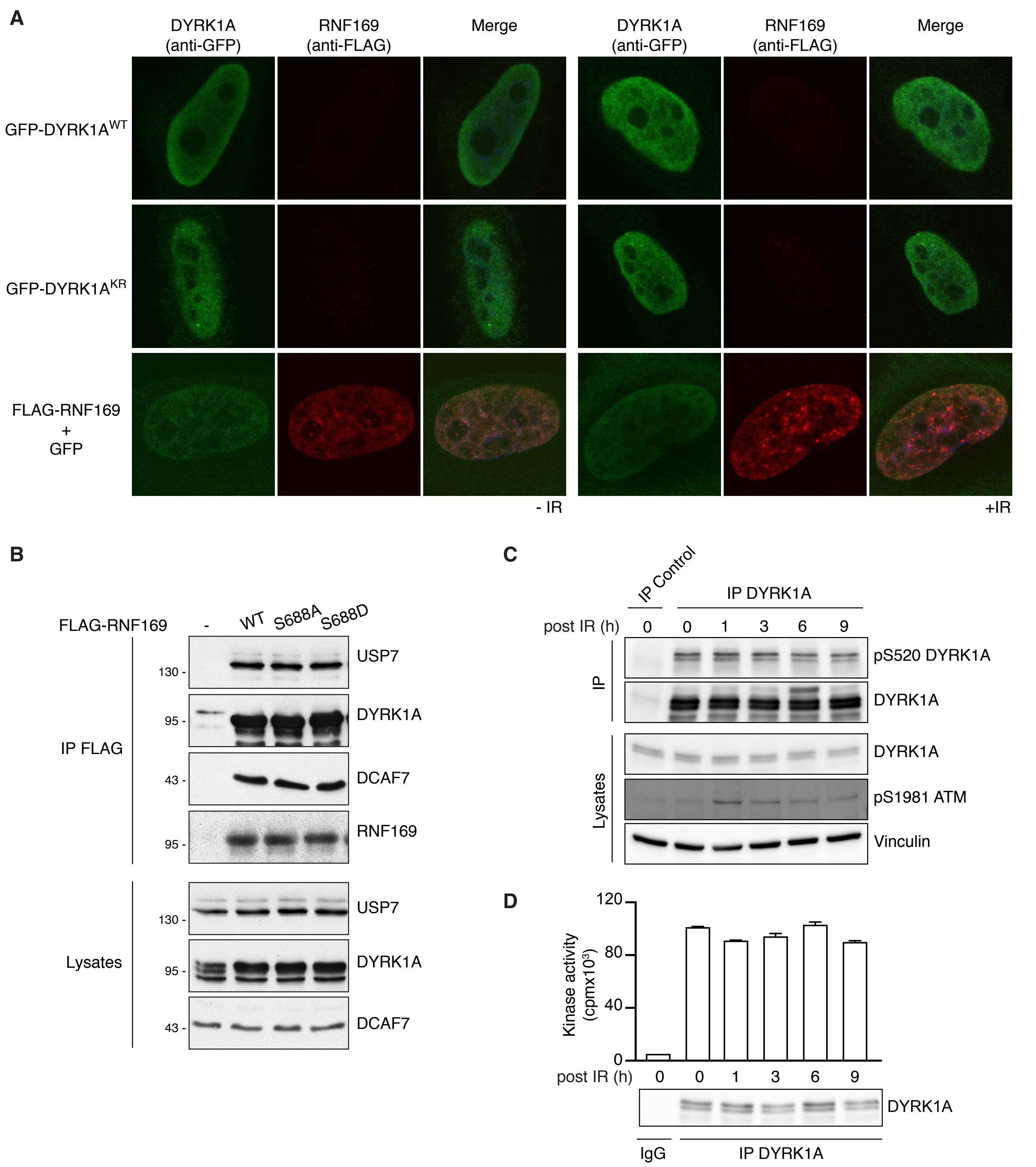
A
DYRK1A (anti-GFP)
RNF169 (anti-FLAG)
Merge
DYRK1A (anti-GFP)
RNF169 (anti-FLAG)
Merge
GFP-DYRK1A^WT
GFP-DYRK1A^KR
FLAG-RNF169 + GFP
- IR
+IR
B
FLAG-RNF169
-
WT
S688A
S688D
IP FLAG
Lysates
130 -
95 -
43 -
95 -
130 -
95 -
43 -
USP7
DYRK1A
DCAF7
RNF169
USP7
DYRK1A
DCAF7
C
IP Control
IP DYRK1A
post IR (h)
0
0
1
3
6
9
IP
Lysates
pS520 DYRK1A
DYRK1A
DYRK1A
pS1981 ATM
Vinculin
D
Kinase activity (cpmx10³)
120
80
40
0
post IR (h)
0
0
1
3
6
9
DYRK1A
IgG
IP DYRK1A

A

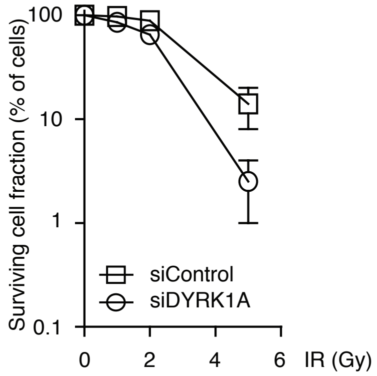
100
10
1
0.1
Surviving cell fraction (% of cells)
siControl
siDYRK1A
0
2
4
6
IR (Gy)

B

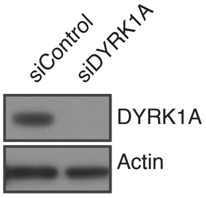
siControl
siDYRK1A
DYRK1A
Actin
